# Variability in newborn telomere length is explained by inheritance and intrauterine environment

**DOI:** 10.1186/s12916-021-02217-9

**Published:** 2022-01-25

**Authors:** Li Chen, Karen Tan Mei Ling, Min Gong, Mary F. F. Chong, Kok Hian Tan, Yap Seng Chong, Michael J. Meaney, Peter D. Gluckman, Johan G. Eriksson, Neerja Karnani

**Affiliations:** 1 Singapore Institute for Clinical Sciences, A*STAR, Singapore, Singapore; 2 Saw Swee Hock School of Public Health, National University of Singapore (NUS), Singapore, Singapore; 3 KK Women’s and Children’s Hospital, Singapore, Singapore; 4 Department of Obstetrics and Gynaecology and Human Potential Translational Research Programme, Yong Loo Lin School of Medicine, National University of Singapore, Singapore, Singapore; 5 Sackler Program for Epigenetics & Psychobiology at McGill University, Montréal, Canada; 6 Ludmer Centre for Neuroinformatics and Mental Health, Douglas Mental Health University Institute, McGill University, Montréal, Canada; 7 Centre for Human Evolution, Adaptation and Disease, Liggins Institute, University of Auckland, Auckland, New Zealand; 8 Folkhalsan Research Center, Helsinki, Finland; 9 Department of General Practice and Primary Health Care, University of Helsinki, Helsinki, Finland; 10 Bioinformatics Institute, A*STAR, Singapore, Singapore; 11 Department of Biochemistry, Yong Loo Lin School of Medicine, National University of Singapore, Singapore, Singapore

**Keywords:** Newborn, Telomere length, Intrauterine exposures, Inheritance, Sex differences

## Abstract

**Background:**

Telomere length (TL) and its attrition are important indicators of physiological stress and biological aging and hence may vary among individuals of the same age. This variation is apparent even in newborns, suggesting potential effects of parental factors and the intrauterine environment on TL of the growing fetus.

**Methods:**

Average relative TLs of newborns (cord tissue, *N* = 950) and mothers (buffy coat collected at 26–28 weeks of gestation, *N* = 892) were measured in a birth cohort. This study provides a comprehensive analysis of the effects of heritable factors, socioeconomic status, and in utero exposures linked with maternal nutrition, cardiometabolic health, and mental well-being on the newborn TL. The association between maternal TL and antenatal maternal health was also studied.

**Results:**

Longer maternal TL (*β* = 0.14, *P* = 1.99E−05) and higher paternal age (*β* = 0.10, *P* = 3.73E−03) were positively associated with newborn TL. Genome-wide association studies on newborn and maternal TLs identified 6 genetic variants in a strong linkage disequilibrium on chromosome 3q26.2 (Tag SNP-*LRRC34*-rs10936600: *P*_meta_ = 5.95E−08). Mothers with higher anxiety scores, elevated fasting blood glucose, lower plasma insulin-like growth factor-binding protein 3 and vitamin B12 levels, and active smoking status during pregnancy showed a higher risk of giving birth to offspring with shorter TL. There were sex-related differences in the factors explaining newborn TL variation. Variation in female newborn TL was best explained by maternal TL, mental health, and plasma vitamin B12 levels, while that in male newborn TL was best explained by paternal age, maternal education, and metabolic health. Mother’s TL was associated with her own metabolic health and nutrient status, which may have transgenerational effects on offspring TL.

**Conclusions:**

Our findings provide a comprehensive understanding of the heritable and environmental factors and their relative contributions to the initial setting of TL and programing of longevity in early life. This study provides valuable insights for preventing in utero telomere attrition by improving the antenatal health of mothers via targeting the modifiable factors.

**Trial registration:**

ClinicalTrials.gov, NCT01174875. Registered on 1 July 2010

**Supplementary Information:**

The online version contains supplementary material available at 10.1186/s12916-021-02217-9.

## Background

Telomeres are nucleoprotein structures formed of non-coding tandem repeats of hexamer TTAGGG at the end of the chromosomes. Functionally, telomeres play an important role in maintaining genomic stability and protect chromosomes from end-to-end fusion and degradation [1]. Telomeres shorten with each cell division, reflecting the age of a cell and the time until senescence [1]. Telomere (TL) is a biomarker of biological aging and has been associated with physiological and environmental stress, age-related diseases, and early mortality [2, 3]. Sex differences have also been reported in multiple studies with females having longer TL than males [4]. Shortened leukocyte TL has been associated with smoking [5], type 2 diabetes [6], higher 2-h post-load glucose concentration [7], lower insulin-like growth factor 1 [8], and higher leptin [9] and homocysteine levels [10]. Multiple studies reported telomere shortening to be associated with hypertension [11], cardiovascular disease [12], and mental disorders [13]. Some studies reported diet [14], parity [15, 16], and educational attainment [17] to explain inter-individual variation in TL.

In addition to diet, lifestyle, and other environmental exposures, heritable factors such as genetic variants have also been recognized to play a significant role in determining an individual’s TL. Genome-wide association studies (GWAS) have been performed on TL in many adult studies, which have identified TL-linked genetic variants at multiple loci in the human genome: *TERC* (3q26.2) [18–22], *TERT* (5q15.33) [20, 21, 23], *RTEL1* (20q13.33) [21, 24], *OBFC1* (10q24.3) [20, 21], *NAF1* (4q32.2) [21], *ZNF208* (19p12) [21], and *ACYP2* (2p16.2) [21].

Newborn TL is the initial setting of TL and highly variable among individuals [25]. It has important implications on telomere dynamics and molecular longevity over the lifespan [26, 27]. Current advances in newborn TL research indicate plasticity in the programming of telomere biology and the initial setting of TL [28]. The in utero environment has been suggested to be a significant contributor to this effect. Cord blood TL association studies with specific in utero exposures such as maternal nutrient status (e.g., folate [29] and vitamin D [30]), smoking status [31], educational attainment [32], or metabolic [33–35] and mental health [36] have provided valuable insights. Offspring TL has also been reported to associate with parental age and TL [37–41]. However, as the existing studies have primarily focused on individual exposures, a comprehensive understanding of the magnitude of their independent effects on offspring TL is lacking in the field. Also, though leukocyte TL in newborn is known to differ by sex, the sexual dimorphism in the factors explaining newborn TL variation is underexplored.

This study provides the comprehensive analysis of the effects of heritable factors, socioeconomic status, and in utero exposures linked with maternal nutrition, cardiometabolic health, and mental well-being on the newborn TL. Sex-specific effects and the contributions of factors to variation in newborn TL were assessed. This comprehensive analysis was feasible due to the availability of a large sample size (*N* = 950) with deep phenotyping and genomics data generated in the Growing Up in Singapore Towards healthy Outcomes (GUSTO) birth cohort.

## Methods

### Study population

We used data from the Growing Up in Singapore Towards healthy Outcomes (GUSTO) study, which is a prospective mother-offspring birth cohort study designed for investigating developmental origins of health and disease (DOHaD). The GUSTO study recruited 1247 pregnant women in their first trimester of pregnancy from two major public hospitals in Singapore, KK Women’s and Children’s Hospital (KKH) and National University Hospital (NUH), between June 2009 and September 2010 [42]. It conducted extensive maternal assessments at 26–28 weeks of gestation and assessments of offspring development and behavior from birth onwards. Participants could be of Chinese, Malay, or Indian ethnicity, but with homogeneous parental ethnic backgrounds.

### Tissue collection and DNA extraction

#### Umbilical cord

Detailed information of collection and processing for the cord tissue has been described previously [43]. Briefly, umbilical cord tissue samples were collected after the extraction of cord blood and then cleaned with phosphate-buffered saline solution. The cord samples were snap-frozen in liquid nitrogen and stored at − 80 °C until subsequent DNA extraction. Before DNA extraction, frozen umbilical cords were pulverized and treated with 10 U/mL hydraluronidase enzyme and then incubated at 37 °C for 30 min. The tissue was homogenized using a Xiril Dispomix homogenizer after adding 250 μL of Tris-NaCl-EDTA-SDS solution. Samples were pulse spun to pellet the tissue prior to adding proteinase K, and incubated overnight at 55 °C. DNA extraction from the lysates was performed using the QIAsymphony Midi DNA kit (QIAGEN), as per the manufacturer’s instructions. Genomic DNA integrity of DNA samples was evaluated by agarose gel electrophoresis to ensure no apparent DNA degradation (Fig. S1A).

#### Maternal blood

Up to 20 mL of blood was collected from the peripheral vein of pregnant women at 26–28 weeks of gestation into EDTA tubes. Blood samples were then centrifuged within 4 h at 1600 g at 4 °C for 10 min to separate the blood into three distinct layers—plasma, buffy coat, and erythrocytes. The top plasma layer was then carefully extracted (without disturbing the buffy coat), followed by extraction of the buffy coat layer. The buffy coat was stored at − 80 °C. DNA extraction from the buffy coat was performed using either the QIAsymphony Midi DNA kit (QIAGEN) as per the manufacturer’s instructions or phenol/chloroform extraction. Briefly, an equal volume of phenol/chloroform was added to the buffy coat and mixed, followed by centrifugation at 13,200 rpm for 10 min. DNA was precipitated from the top aqueous layer using 1/10th volume of 3 M NaAc and 2 volumes of ice-cold 100% ethanol. The DNA pellet was obtained by centrifugation at 13,200 rpm for 10 min and air-dried at 60 °C before dissolving in double-distilled water. Genomic DNA integrity of DNA samples was evaluated by agarose gel electrophoresis to ensure no apparent DNA degradation (Fig. S1B).

#### Sample/DNA procedures

Cord tissue and maternal blood samples were collected within 1.5 years. Sample collection month was divided into four quarters in cord tissue samples (January to March, 33.8%; April to June, 16.3%; July to September, 21.6%; and October to December, 28.3%) and maternal blood samples (January to March, 18.5%; April to June, 20.3%; July to September, 27.0%; and October to December, 34.2%). Sample storage time was varied from 4~6 years for cord tissue samples and 3~4 years for maternal blood samples. DNA storage time (− 80 °C freezer) was varied from 0~2 years (81.3%, < 1 year, and 18.7%, ~ 2 years) for cord tissue and 3~4 years (17.8%, ~ 4 years, and 82.2%, ~ 3 years) for maternal blood samples.

### Average relative telomere length measurement

Average relative TLs of cord tissue and maternal buffy coat samples were measured by the Blackburn Laboratory (https://blackburnlab.ucsf.edu). Cord tissue and maternal samples were measured separately and run as two batches, using the same reagent lots for each batch. They were measured by a modified quantitative real-time PCR (qPCR) protocol which has been described previously in detail [44]. Briefly, this method generates a measure of the average TL of each DNA sample as a ratio (T/S) of telomere repeat length (T) to the copy number of a single-copy gene (S, human beta-globin). All PCRs were performed on a Roche LightCycler 480 real-time PCR machine with 384-well capacity. Eight control DNA samples were included in each run to account for inter-assay variability. The T/S ratio of each control DNA was divided by the average T/S for the same DNA from 10 runs to obtain a normalizing factor. This procedure was implemented for all eight control samples, and then the average normalizing factor was used to correct the participant DNA samples to obtain the final T/S ratio. Each sample was measured twice for the T/S ratio. But if the difference between the two measurements varied by more than 7%, the sample was run a third time and then the average of the two closest values was reported. The above method was used for measuring TLs of newborn and maternal DNA samples. Nine assay plates were used for cord tissue samples, and ten assay plates were used for maternal samples. The inter-assay coefficient of variant (CV) for newborn TL was 2.5%, and the CV for maternal TL was 2.7%. The intra-class correlation coefficient (ICC) [45] was 0.924 (95%CI 0.913–0.934) for newborn TL and 0.978 (95%CI 0.975–0.980) for maternal TL (Additional file 1: Table S1).

A pilot study was conducted for the comparison of the average relative TL of cord blood and cord tissue using 20 subjects.

### Genotype data

Both newborn and maternal DNA samples were genotyped on Illumina HumanOmniExpressExome arrays. Samples with a call rate less than 97%, cryptic relatedness, or sex/ethnic discrepancies were excluded. SNPs with call rates less than 95% or minor allele frequency less than 5% or Hardy-Weinberg equilibrium *P* value less than 1.00E−06 and non-autosomal SNPs were excluded.

### Newborn sex, birth weight, gestational age, and paternal age

Newborn sex and birth weight were extracted from the medical records. Gestational age (GA) was assessed by ultrasonography by trained ultrasonographers. GA was first assessed during the first trimester of pregnancy and reported in completed weeks. Paternal age was collected at recruitment by interviewer-administered questionnaires. Five paternal subjects with age > 55 years were excluded from the analysis.

### Maternal characteristics during pregnancy

#### Demographic and anthropometric data

At enrollment, interviewer-administered questionnaires were used to collect information on age, ethnicity (Chinese, Malay, and Indian), educational attainment (secondary and below, post-secondary, and university), monthly household income (≤ S$1999, 2000–5999, and ≥ 6000), and pre-pregnancy weight. Parity (primiparous and multiparous) was extracted from hospital medical records.

Weight and height were measured at 26–28 weeks of gestation. Gestational weight gain (GWG) was calculated as the difference between pre-pregnancy weight and weight at 26–28 weeks’ gestation. Pre-pregnancy body mass index (ppBMI) was calculated as pre-pregnancy weight divided by height squared. Peripheral systolic pressure and diastolic blood pressure were measured from the brachial artery at 26–28 weeks’ gestation.

#### Maternal mental health

Information on depressive and anxiety symptoms was obtained by questionnaires at 26–28 weeks of gestation. The Edinburgh Postnatal Depression Scale (EPDS) was used to assess the depressive symptoms by 10 items of common depressive symptoms over the past week. Anxiety was assessed by the Spielberger State-Trait Anxiety Inventory (STAI), which consists of 40 items with a 4-point Likert scale. Twenty items assess the state measure which reflects transient characteristics of anxiety (i.e., anxiety disorders), while the other twenty items were used to assess the trait measure, reflecting a more stable personality characteristic, such as an anxious personality.

#### Maternal plasma glucose concentration

The participants underwent an oral glucose tolerance test (OGTT) at the 26th–28th week of pregnancy visit [46]. Fasting plasma glucose concentrations (FPG) were measured after an overnight fasting (8–14 h), and 2-h post-load glucose concentrations (2-h PG) were measured at 2 h after taking 75 g of glucose. Gestational diabetes mellitus (GDM) status was diagnosed on the basis of the World Health Organization 1999 criteria: ≥ 7.0 mmol/L for FPG and/or ≥ 7.8 mmol/L for 2-h PG [47]. Three subjects were excluded with FPG> = 7.0 mmol/L since they were diabetic. In this study, all GDM cases are diagnosed based on the cutoff of 2-h post-load glucose concentrations.

#### Maternal plasma fatty acids, vitamins, metabolites, and biomarkers

Blood was drawn at 26–28 weeks’ gestation into EDTA tubes. Blood samples were centrifuged at 1600 g at 4 °C for 10 min within 4 h and stored at − 80 °C prior to analysis. Plasma fatty acids, vitamins, metabolites, and biomarkers were measured as reported previously [48–52].

Plasma phosphatidylcholine (PC) fatty acids were measured, and the fatty acids were expressed in percentage of total fatty acids in plasma PC. We investigated the total saturated fatty acids (SFA), the total mono-unsaturated fatty acids (MUFA), the total n-3 poly-unsaturated fatty acids (PUFA), the total n-6 PUFA, and the ratio of them (n-6:n-3 PUFA). We also investigated the top three abundant fatty acids in n-3 PUFA (docosahexaenoic acid (DHA), docosapentaenoic acid (DPA), and eicosapentaenoic acid (EPA)) and n-6 PUFA (linoleic acid (LA), dihomo-gamma-linolenic acid (DGLA), and arachidonic acid (AA)).

Vitamin B6, betaine, choline, and total homocysteine concentrations were measured by liquid chromatography-tandem mass spectrometry (LC-MS/MS) at BEVITAL AS (http://www.bevital.no). Folate and vitamin B12 concentrations were measured using the ADVIA Centaur Immunoassay System. 25-Hydroxy vitamin D_3_ was analyzed by isotope-dilution liquid chromatography-tandem mass spectrometry (ID-LC-MS/MS).

Protein biomarkers were analyzed by MILLIPLEX® Multiplex Assays Using Luminex®. We investigated adiponectin, leptin, C-reactive protein (CRP), plasminogen activator inhibitor-1 (PAI-1), insulin-like growth factors (IGF1 and IGF2), and insulin-like growth factor binding proteins (IGFBP1, IGFBP3, IGFBP4, and IGFBP7).

#### Maternal smoking and drinking

Maternal alcohol consumption before and during pregnancy and maternal smoking status before and during pregnancy were extracted from an interviewer-administered questionnaire at 26–28 weeks’ gestation. Plasma cotinine concentration was measured by LC-MS/MS from blood samples drawn at 26–28 weeks’ gestation. In order to avoid underreported smoking during pregnancy, a combined method was applied using self-reported smoking status and plasma cotinine level. Smoking status was derived when self-reported status is smoking or cotinine level is greater than 10 ng/mL [53]. Non-smoking status was identified by self-reported non-smoking and cotinine level ≤ 10 ng/mL.

### Statistical analysis

After evaluating DNA quality and PCR performances of TL measurement, the selection criteria for newborns (APGAR ≥ 9 and singleton), and the availability of genotype data, 950 subjects were included in this study as they had their newborn TL measured with complete information on sex, ethnicity, maternal age, gestational age, and genotype data. The basic characteristics of these 950 newborns were not significantly different from those of a total of 1177 live singleton newborns in the cohort (Additional file 1: Table S2). A subset of participants had available paternal age, maternal TL, and maternal characteristics during pregnancy as shown in Additional file 1: Table S3. Normality was checked for all the continuous variables. As the distributions of some continuous variables were skewed, we did log10 transformation and then the values were truncated to the nearest possible value if they were > 4 SDs from the mean.

#### Genome-wide association studies (GWAS)

Trans-ethnic GWAS were applied on newborn TL (*N* = 950) and maternal TL (*N* = 892). For newborn or maternal genotype data, we did SNP quality control processing in each ethnicity. The SNPs that passed the quality control in at least one ethnicity were used for trans-ethnic GWAS on newborn TL (615,105 SNPs) and maternal TL (613,953 SNPs). The genotypes of each genetic variant were coded as 0-AA, 1-Aa, and 2-aa using an additive model (A, reference allele; a, effect allele). The coded genotype data and *z*-scores of newborn/maternal TL were used in regression analysis. The effect size indicates the SD difference in TL per dosage change of effect allele. Sex and ethnicity were adjusted in newborn trans-ethnic GWAS (*N* = 950), while ethnicity and maternal age were adjusted in maternal trans-ethnic GWAS (*N* = 892). A meta-analysis of two studies was carried out using the inverse-variance-based approach (609,579 SNPs). As a measure for heterogeneity between studies, the fraction of variation across studies (*I*^2^) that is due to heterogeneity rather than chance and *P* value for the heterogeneity test (*P*_het_) were calculated. GWAS and meta-analysis were performed in PLINK 1.9.

#### Association with newborn TL

Linear regression was used to study the association between newborn TL (outcome) and clinical variables (predictors) using univariate and multivariate analyses. For continuous predictors, *z*-scores of outcome and predictor variables were used in regression analysis, and then the standardized effect sizes *β* (SD/SD) were reported in the results. For categorical (i.e., ethnicity, GDM status, smoking status, alcohol assumption, and parity) and ordinal (i.e., education and household income) predictors, only *z*-scores of the outcome variable were used in regression analysis, and then the effect sizes *β* (SD difference between the groups) were reported in the results. DNA extraction for all umbilical cord samples was performed using the same method. Sex and ethnicity were adjusted in multivariate analysis. Furthermore, sex stratification analysis was performed after adjustment for ethnicity. The association between newborn TL and influencing factors was examined separately in male and female newborns. The subjects with missing values were excluded in the analysis of each factor.

#### Association with maternal TL

The association study between maternal TL and antenatal maternal health was examined by linear regression. As maternal age, ethnicity, and DNA extraction methods of maternal blood samples were significantly associated with maternal TL, they were adjusted in the regression models. Two multivariate models were implemented to assess the significance of each factor after accounting for the effects of (1) maternal age, ethnicity, and DNA extraction method and (2) maternal age, ethnicity, DNA extraction method, and GDM status. The subjects with missing values were excluded in the analysis of each factor. The calculation of effect sizes was the same as above. The mediating effect of maternal TL was studied for the factors that were significantly associated with both newborn and maternal TLs.

#### Comparative assessment factors associated with newborn TL

In order to check for independent significances of influencing factors and their contributions to variance of newborn TL, twelve significant factors listed in Table 2 were considered in this analysis. Representative variables were selected from parental age and mental health since variables are highly correlated within the same category. For parental ages, paternal age was selected for its dominant effect on newborn TL. For mental health, STAI trait score was selected for representing this category because it exhibited much stronger association than STAI state and EPDS. In consideration of the GWAS result, ten selected factors were examined in three multivariate models using all, female-only, and male-only subjects. They are newborn genetic variant *LRRC34*-rs10936600, maternal TL, paternal age, STAI trait score, fasting glucose, DGLA%, IGFBP3, vitamin B12, maternal education, and smoking status during pregnancy. In the process of model selection, sex and ethnicity were included in the basic model for all subjects, and ethnicity only was used in the basic models for male-only and female-only subjects. The forward selection method was applied to add predictors, and the Akaike information criterion was used to select the best multivariate model, in which sample size was reduced due to the missing values of required predictors.

#### Sensitivity analysis

As sample collection, DNA storage procedures, and seasonality have been reported to be associated with telomere length measurement [54], additional adjustment of these factors in the best multivariate models of newborn TL and the association studies between maternal TL and antenatal factors were performed. Sample storage time is highly correlated to DNA storage time in our study. Hence, only DNA storage time (two groups) and sample collection month (four quarters) were studied in the sensitivity analysis.

The association analyses with newborn TL and maternal TL were performed in MATLAB R2019b. The calculation of intra-class correlation coefficients (rptR package) and mediation analysis of maternal TL (mediation package) were carried out in R 3.6.3.

## Results

### Participant characteristics and TL measures

**Fig. 1:**
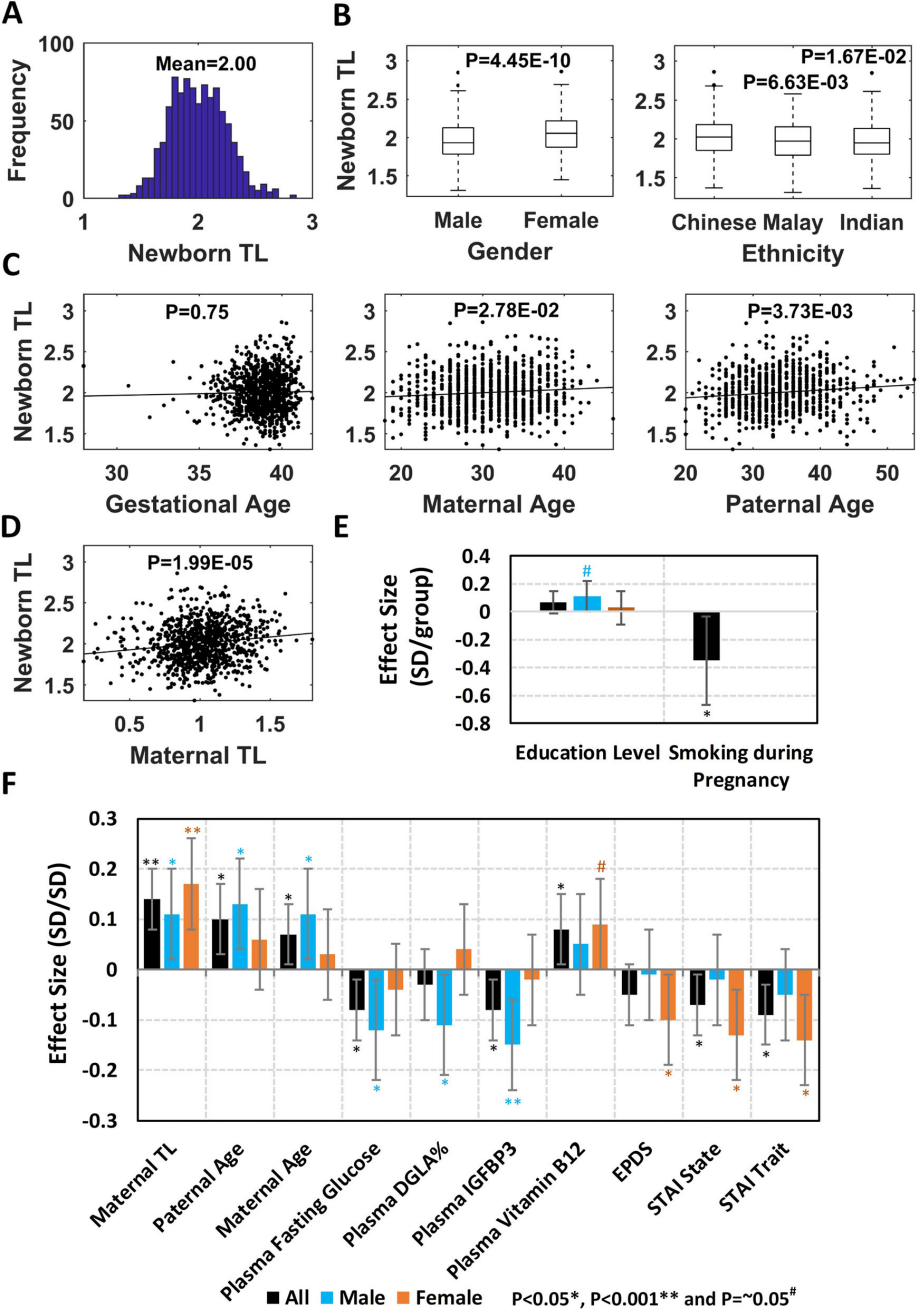
Association studies of newborn telomere length. **A** Histogram of newborn telomere length. **B** Boxplots of sex and ethnicity (univariate *P* values). **C**, **D** Scatter plots of gestational age, maternal age, paternal age, and maternal telomere length (*P* values after adjusting for sex and ethnicity). **E**, **F** Effect size plot of factors significantly associated with newborn telomere length using all, female-only, and male-only subjects (error bar, 95%CI). Related to Table 2. **E** Categorical/ordinal variables. **F** Continuous variables

**Fig. 2:**
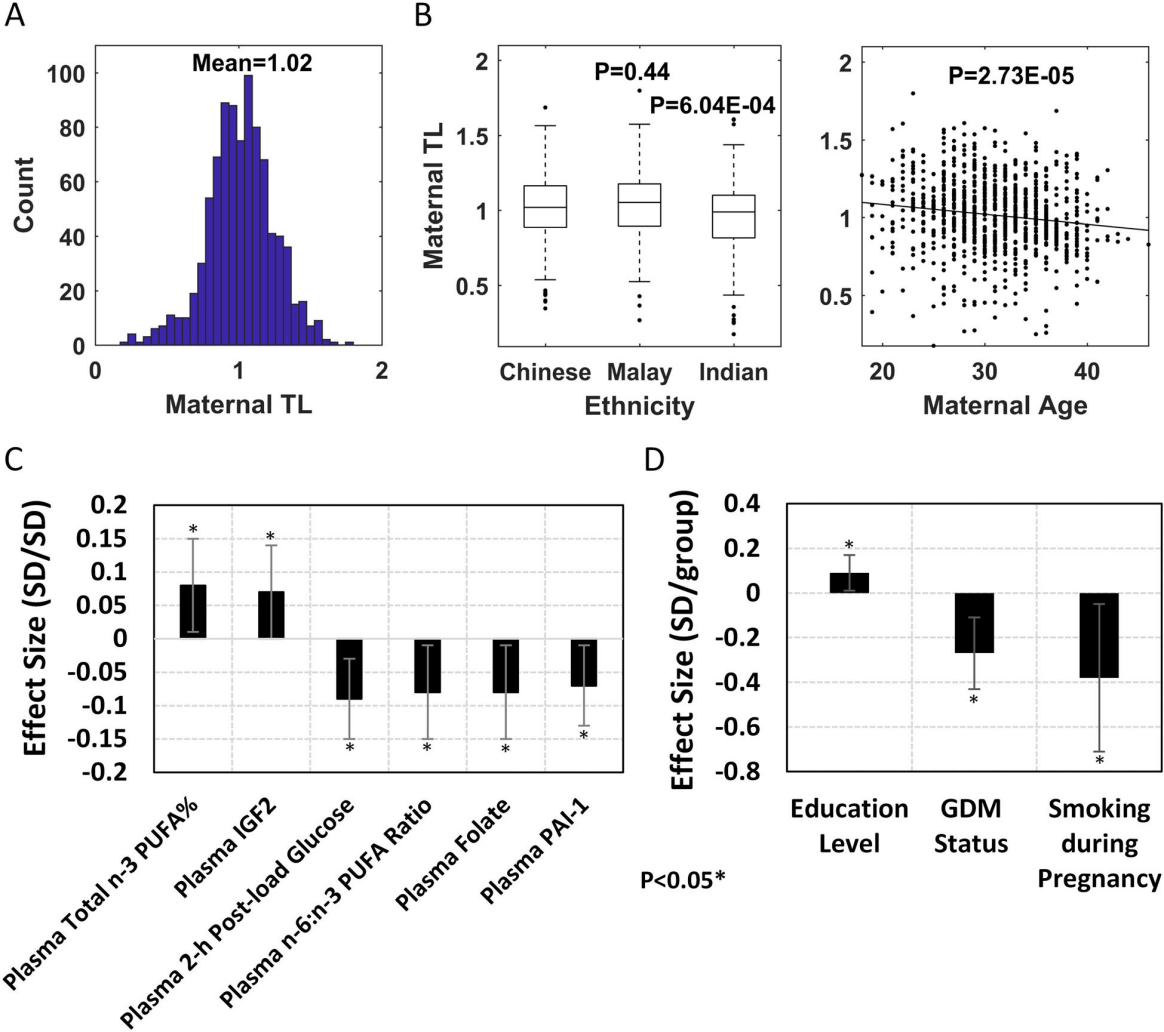
Association studies of maternal telomere length. **A** Histogram of maternal telomere length. **B** Boxplot of ethnicity and scatter plot of maternal age (univariate *P* values). **C**, **D** Effect size plot of significant factors associated with maternal telomere length after adjusting for age, ethnicity, and DNA extraction method (error bar, 95%CI). Related to Additional file 1: Table S6 (main model). **C** Continuous variables. **D** Categorical/ordinal variables

Depending on the availability of key variables required for this study, data from 950 of 1247 mother-offspring dyads were analyzed from the GUSTO cohort. A flow chart of sample selection and data analysis is provided in Fig. S2. Clinical characteristics and TL measurement summary of all participants in this study are shown in Additional file 1: Table S3. These include newborn TL, maternal TL, infant sex, ethnicity, parental age, gestational age, birth weight, maternal smoking and alcohol consumption status, and a wide range of antenatal maternal cardiometabolic, mental health, and plasma nutrient measures. Both newborn and maternal TLs showed normal distribution (Figs. 1A and 2A). The average newborn TL is 2.00 with a standard deviation of 0.25, and the average maternal TL is 1.02 with a standard deviation of 0.23.

### Sex difference and ethnic diversity in newborn TL

Newborns in the study comprised 52.6% males and 47.4% females. Notably, sex was the most significant factor (*β* = 0.40, *P* = 4.45E−10) associated with newborn TL in univariate analysis (Additional file 1: Table S3). The average TL at birth in females (mean = 2.05, SD = 0.24) was longer than that in males (mean = 1.96, SD = 0.24) (Fig. 1B). All mother-offspring dyads in this study were of Asian ethnic origin, i.e., Chinese (58.4%), Malay (24.6%), and Indian (17.0%). Compared to the TL of newborn of Chinese ethnic origin (mean = 2.02, SD = 0.25), TLs of Malay (mean = 1.97, SD = 0.24; *β* = − 0.21, *P* = 6.63E−03) and Indian (mean = 1.97, SD = 0.24; *β* = − 0.21, *P* = 1.67E−02) newborns were slightly shorter (Fig. 1B).

### Paternal age is positively associated with newborn TL

Newborn TL was not associated with gestational age and birth weight (Additional file 1: Table S3). As is evident from Fig. 1C, maternal age (*β* = 0.07, *P* = 2.78E−02) and paternal age (*β* = 0.10, *P* = 3.73E−03) showed a significant positive association with the newborn TL. Since maternal age is highly correlated with the paternal age (*R*^2^ = 0.50) in the GUSTO cohort, we also analyzed them in the same linear regression model with sex and ethnicity as covariates. Interestingly, the association between maternal age and newborn TL was lost in such an analysis, but paternal age remained significant (*β* = 0.14, *P* = 4.22E−03). This finding indicates that among the parents, paternal age has a dominant effect on newborn TL, and hence, offsprings of older fathers are born with longer TL.

### Inheritance/heritability of TL in newborn

To elucidate the factor of heritability in newborn TL, we first examined if maternal TL was a predictor of newborn TL. The correlation analysis confirmed this to be true as the mothers with longer TL gave birth to offsprings with longer TL (Fig. 1D; *β* = 0.14, *P* = 1.99E−05).

We next examined if the heritability of TL was influenced by genetics via performing genome-wide association studies (GWAS) of newborn and maternal TLs. Though no SNPs passed the genome-wide significance cutoff (*P* = 5.00E−08) in the individual GWASs (Fig. S3A-B), a meta-analysis of these two studies (Fig. S3C) revealed top six genetic variants (*LRRC34*-rs10936600, rs13069553, *LRRC34*-rs7621631, *MYNN*-rs1317082, *MYNN*-rs10936599, and rs12638862) within the same genomic region 3q26.2 (Table 1 and Additional file 1: Table S4). The top variant *LRRC34*-rs10936600 (missense) showed a borderline genome-wide significance (*P* = 5.95E−08; *β* = − 0.18). Locus zoom plot using the 1000 genome ASN population (Fig. S3D) identified these six genetic variants to be in a strong linkage disequilibrium (LD) with each other. Their pairwise *R*^2^ in three ethnic groups are provided in Additional file 1: Table S5 (*R*^2^ ≥ 0.82). Among these six SNPs, *LRRC34*-rs10936600 constituted the tag SNP of this LD block. This 3q26.2 genomic region has previously been reported to be associated with TL in adults [18, 19]. Notably, rs12638862 is located 4891 bp downstream of the *TERC* (telomerase RNA component) gene, which is known to be essential for telomere length maintenance. All six variants showed no observed heterogeneity between maternal and newborn genotype data (*P*_het_ > 0.1 and *I*^2^ = 0).

**Table 1:** Top six genetic variants (3q26.2) in the meta-analysis results of genome-wide association studies on newborn telomere length and maternal telomere length

SNP	Chr:position^#^	Allele R/E	Gene	Newborn telomere length (*N* = 950)		Maternal telomere length (*N* = 892)		Meta-analysis			
				*β* (SE)	*P* value	*β* (SE)	*P* value	*β* (SE)	*P* value	*P*_het_	*I*^2^
rs10936600	3:169514585	A/T^*^	*LRRC34*^a^	− 0.20 (0.05)	2.35E−05	− 0.16 (0.05)	6.79E−04	− 0.18 (0.03)	5.95E−08	0.58	0
rs13069553	3:169508272	A/G	Intergenic^b^	− 0.20 (0.05)	1.57E−05	− 0.16 (0.05)	9.54E−04	− 0.18 (0.03)	6.00E−08	0.50	0
rs7621631	3:169512145	C/A	*LRRC34*^c^	− 0.20 (0.05)	2.00E−05	− 0.16 (0.05)	8.30E−04	− 0.18 (0.03)	6.35E−08	0.54	0
rs1317082	3:169497585	A/G	*MYNN*^d^	− 0.20 (0.05)	1.76E−05	− 0.16 (0.05)	9.69E−04	− 0.18 (0.03)	6.79E−08	0.50	0
rs10936599	3:169492101	G/A	*MYNN*^e^	− 0.19 (0.05)	3.93E−05	− 0.16 (0.05)	9.15E−04	− 0.18 (0.03)	1.32E−07	0.59	0
rs12638862	3:169477506	G/A	Intergenic^f^	0.16 (0.05)	5.86E−04	0.15 (0.05)	1.18E−03	0.16 (0.03)	2.14E−06	0.91	0

*P*_het_ > 0.1 and *I*^2^ = 0 showed no observed heterogeneity

*R/E* reference/effect allele, *β (SE)* effect size (standard error)

^#^hg19 genome build

*Forward strand

^a^Missense L286I

^b^767 bp downstream of *MYNN*

^c^Intron variant, 3′ UTR variant

^d^Intron variant

^e^Synonymous codon

^f^4891 bp downstream of *TERC*

Ethnicity-specific box plots for each of the SNPs and their allele frequencies are provided in Fig. S4-S5. It was noted that the dosage of A allele of the top variant *LRRC34*-rs10936600 was positively associated with TL. Furthermore, sex stratification results on newborn *LRRC34*-rs10936600 showed that its effect on newborn TL was much stronger in males (*β* = − 0.23, *P* = 4.46E−04) than in females (*β* = − 0.16, *P* = 1.59E−02).

### Maternal antenatal health, smoking, and nutrient status have a significant impact on newborn TL

Since the variability in TL can arise from both genetic and environmental factors, we next studied the effects of maternal antenatal health, blood nutrient levels, and socioeconomic status on newborn TL. These variables included mother’s mental health, adiposity, blood pressure, plasma glucose concentration, plasma fatty acids and vitamins, plasma protein biomarkers, educational attainment and household income, smoking status, alcohol consumption, and parity. Heat map of pairwise Pearson correlation coefficients between these clinical variables (continuous measures) is provided in Fig. S6. The results of univariate and multivariate regression analyses for these variables are provided in Additional file 1: Table S3.

The link between newborn TL and antenatal mental health was studied using the Edinburgh Postnatal Depression Scale (EPDS) and the State-Trait Anxiety Inventory (STAI) scores collected during mid-pregnancy (26–28 weeks). While EPDS scores showed no significant association with newborn TL, STAI scores showed strong negative correlation with newborn TL, indicating higher antenatal anxiety to be associated with shorter newborn TL. Among STAI measures, STAI trait scores (*β* = − 0.09, *P* = 3.61E−03) were more significantly associated with newborn TL than STAI state scores (*β* = − 0.07, *P* = 2.93E−02).

For cardiometabolic health measures, maternal adiposity (ppBMI, GWG, and height), blood pressure, plasma protein biomarkers, and antenatal glycemia (fasting glucose, 2-h post-load glucose concentrations, and GDM status) were investigated for their associations with newborn TL. Only fasting glucose (*β* = − 0.08, *P* = 1.60E−02) and insulin-like growth factor-binding protein 3 (IGFBP3) (*β* = − 0.08, *P* = 7.62E−03) demonstrated significant negative associations with newborn TL.

As maternal nutrition plays a critical role in fetal development, we also investigated the effects of antenatal plasma fatty acids and vitamin levels on newborn TL. Percentages of ten fatty acids and n-6:n-3 PUFA ratio in antenatal plasma were assessed, but none of them showed significant association. Among the vitamins tested, vitamin B12 level in gestation showed a significant positive association with newborn TL (*β* = 0.08, *P* = 2.87E−02), and vitamin D_3_ level was significant only in the univariate analysis.

Two factors describing the socioeconomic status (SES) were evaluated, i.e., household income and maternal education. Both were significant only in the univariate analysis. As smoking is a well-known factor in shortening of TL, maternal smoking before and during pregnancy was assessed. Smoking status before pregnancy was not significant while maternal smoking during pregnancy (39 of 827 mothers are smokers) showed stronger effects on the newborn TL (*β* = − 0.35, *P* = 3.06E−02). The effects of alcohol consumption before and during pregnancy on newborn TL were not significant; however, the outcomes from such an analysis should be interpreted with caution as the sample size (*N* = 21) of mothers consuming alcohol during pregnancy was very small in the GUSTO cohort. Lastly, parity (primiparous and multiparous) was explored, but it had no significant effects on newborn TL.

From the above analyses, we concluded that a women’s mental well-being, metabolic health, and nutrient and smoking status during pregnancy have significant effects on newborn TL. Figure 1E, F provides a comparison of the effect sizes of these factors.

### Sex-specific effects of factors influencing newborn TL

In order to study the sex difference in newborn TL, both stratification and interaction analyses were performed. Stratification analysis studied sex-specific effects of factors influencing newborn TL. Table 2 summarizes the significant factors derived from multivariate regression analyses using either all, or female-only, or male-only newborns.

**Table 2:** Linear regression results between newborn telomere length and significant factors using all, male-only, and female-only subjects

Variable	Multivariate model (adjusted for sex and ethnicity), all			Multivariate model (adjusted for ethnicity), male			Multivariate model (adjusted for ethnicity), female		
	*N*	*β* (95%CI)	*P* value	*N*	*β* (95%CI)	*P* value	*N*	*β* (95%CI)	*P* value
Maternal telomere length (T/S)	892	0.14 (0.08, 0.20)	**1.99E**−05**	465	0.11 (0.02, 0.2)	**1.67E**−02*	427	0.17 (0.08, 0.26)	**3.09E**−04**
Paternal age (years)	805	0.10 (0.03, 0.17)	**3.73E**−03*	427	0.13 (0.04, 0.22)	**3.53E**−03*	378	0.06 (− 0.05, 0.16)	2.83E−01
Maternal age (years)	950	0.07 (0.01, 0.14)	**2.78E**−02*	500	0.11 (0.02, 0.20)	**1.60E**−02*	450	0.03 (− 0.06, 0.12)	5.36E−01
EPDS	918	− 0.05 (− 0.11, 0.02)	1.40E−01	484	− 0.01 (− 0.1, 0.07)	7.77E−01	434	− 0.10 (− 0.19, 0.00)	**4.23E**−02*
STAI State Score	895	− 0.07 (− 0.14, − 0.01)	**2.93E**−02*	470	− 0.02 (− 0.11, 0.08)	7.37E−01	425	− 0.13 (− 0.22, − 0.04)	**3.79E**−03*
STAI Trait Score	891	− 0.09 (− 0.16, − 0.03)	**3.61E**−03*	468	− 0.05 (− 0.15, 0.04)	2.54E−01	423	− 0.14 (− 0.22, − 0.05)	**2.22E**−03*
Plasma fasting glucose (mmol/L)	905	− 0.08 (− 0.14, − 0.01)	**1.60E**−02*	476	− 0.12 (− 0.22, − 0.02)	**1.39E**−02*	429	− 0.04 (− 0.13, 0.04)	3.21E−01
Plasma DGLA%	831	− 0.03 (− 0.09, 0.04)	4.65E−01	432	− 0.11 (− 0.22, − 0.01)	**3.04E**−02*	399	0.04 (− 0.05, 0.13)	3.28E−01
Plasma IGFBP3 (ng/mL), log_10_	938	− 0.08 (− 0.15, − 0.02)	**7.62E**−03*	493	− 0.15 (− 0.23, − 0.06)	**8.29E**−04**	445	− 0.02 (− 0.11, 0.07)	7.09E−01
Plasma vitamin B12 (pg/mL), log_10_	834	0.08 (0.01, 0.14)	**2.87E**−02*	435	0.05 (− 0.04, 0.15)	2.80E−01	399	0.09 (0.00, 0.19)	5.39E−02
Maternal education									
1: Secondary and below	283	0.07 (− 0.01, 0.16)	7.79E−02	154	0.11 (0.00, 0.23)	5.28E−02	129	0.03 (− 0.09, 0.15)	6.55E−01
2: Post-secondary	336			172			164		
3: University	319			166			153		
Maternal Smoking during Pregnancy									
0: no	788	− 0.35 (− 0.67, − 0.03)	**3.06E**−02*	–	–	–	–	–	–
1: yes	39								

The details of characteristics and the univariate and multivariate analysis results of all factors were provided in Additional file 1: Table S3

Underlined data indicates borderline *P* value

*β* effect size, *CI* confidence interval, *EPDS* Edinburgh Postnatal Depression Scale, *STAI* State-Trait Anxiety Inventory, *DGLA* dihomo-gamma-linolenic acid, *IGFBP3* insulin-like growth factor-binding protein 3

*P < 0.05 and **P < 0.001

Interestingly, maternal TL was the only factor that showed significant association with TL in both male (*β* = 0.11, *P* = 1.67E−02) and female (*β* = 0.17, *P* = 3.09E−04) newborns, but the effects were much stronger in females. Maternal mental health showed significant negative effects on female newborn TL (EPDS: *β* = − 0.10, *P* = 4.23E−02; STAI state: *β* = − 0.13, *P* = 3.79E−03; STAI trait: *β* = − 0.14, *P* = 2.22E−03) but no significant effects on male newborn TL. Female newborn TL-specific associations were also observed with maternal antenatal plasma vitamin B12 levels (*β* = 0.09, *P* = 5.39E−02). Overall, TL in female newborn was more susceptible to variation in maternal TL, mental health, and vitamin B12 levels (Fig. 1E, F and Fig. S7).

For male newborn TL-specific factors, parental age showed significant positive associations (paternal age: *β* = 0.13, *P* = 3.53E−03; maternal age: *β* = 0.11, *P* = 1.60E−02). Likewise, it was noted that plasma fasting glucose concentration (*β* = − 0.12, *P* = 1.39E−02), DGLA% (*β* = − 0.11, *P* = 3.04E−02) and IGFBP3 level (*β* = − 0.15, *P* = 8.29E−04) exhibited significant negative association with male newborn TL. A borderline significant association with male newborn TL was also found with maternal educational attainment (*β* = 0.11, *P* = 5.28E−02). Taken together, variation in newborn male TL was more explained by their parental age, maternal education, plasma fasting glucose, DGLA%, and IGFBP3 levels (Fig. 1 E, F and Fig. S7).

Association of newborn TL with smoking status before pregnancy did not explain sex differences. We were unable to perform a similar analysis for smoking status during pregnancy, as the sample size was extremely small.

Furthermore, interaction analysis was applied to study the interaction between sex and exposure factors after adjusting for ethnicity. The results revealed that only two factors (plasma DGLA%: *P* = 3.22E−02 and plasma IGFBP3 level: *P* = 3.87E−02) were significant for sex interaction.

### Maternal TL is associated with antenatal metabolic health and the nutrient status

As significant inheritance effect from maternal TL was found on newborn TL, the association studies were performed between maternal TL and antenatal maternal factors. It was observed that maternal age (*β* = − 0.14, *P* = 2.73E−05), ethnicity (Indian vs Chinese: *β* = − 0.31, *P* = 6.04E−04; Malay vs Chinese: *β* = 0.06, *P* = 0.44), and DNA extraction method (*P* < 1.00E−05) strongly affected maternal TL measurement, so they were adjusted in the multivariate analysis (Fig. 2B).

We found nine factors to be significantly associated with maternal TL (Additional file 1: Table S6 and Fig. S8). For antenatal glycemia, plasma 2-h post-load glucose concentration (*β* = − 0.09, *P* = 6.62E−03) and GDM status (*β* = − 0.27, *P* = 1.64E−03) identified significant negative association. For maternal nutrients, total n-3 PUFA% (*β* = 0.08, *P* = 3.23E−02), n-6:n-3 PUFA ratio (*β* = − 0.08, *P* = 2.19E−02), and folate level (*β* = − 0.08, *P* = 2.27E−02) showed strong association with maternal TL. Among plasma protein biomarkers, PAI-1 (*β* = − 0.07, *P* = 2.40E−02) and IGF2 (*β* = 0.07, *P* = 3.25E−02) levels presented significant associations with maternal TL. In addition, maternal educational attainment (*β* = 0.09, *P* = 2.73E−02) and maternal smoking during pregnancy (*β* = − 0.38, *P* = 2.42E−02) were also significantly associated with maternal TL. A comparison of effect sizes for all the antenatal factors associated with maternal TL is shown in Fig. 2C, D.

As GDM status showed the strongest association with maternal TL, an additional model was used to identify the interdependencies between GDM status and other significant factors by adjusting for maternal age, ethnicity, DNA extraction method, and GDM status (Additional file 1: Table S6). Interestingly, most of factors showed consistent significance except plasma folate level. Plasma folate level has been reported to be positively associated with 2-h post-load glucose concentration in the same cohort [55], which may explain the negative association between plasma folate level and maternal TL.

Overall, maternal TL is strongly associated with antenatal factors, especially metabolic health and nutrient status, and consequently, these may have a transgenerational bearing on the offspring’s TL.

### Comparative assessment factors associated with newborn TL

**Fig. 3:**
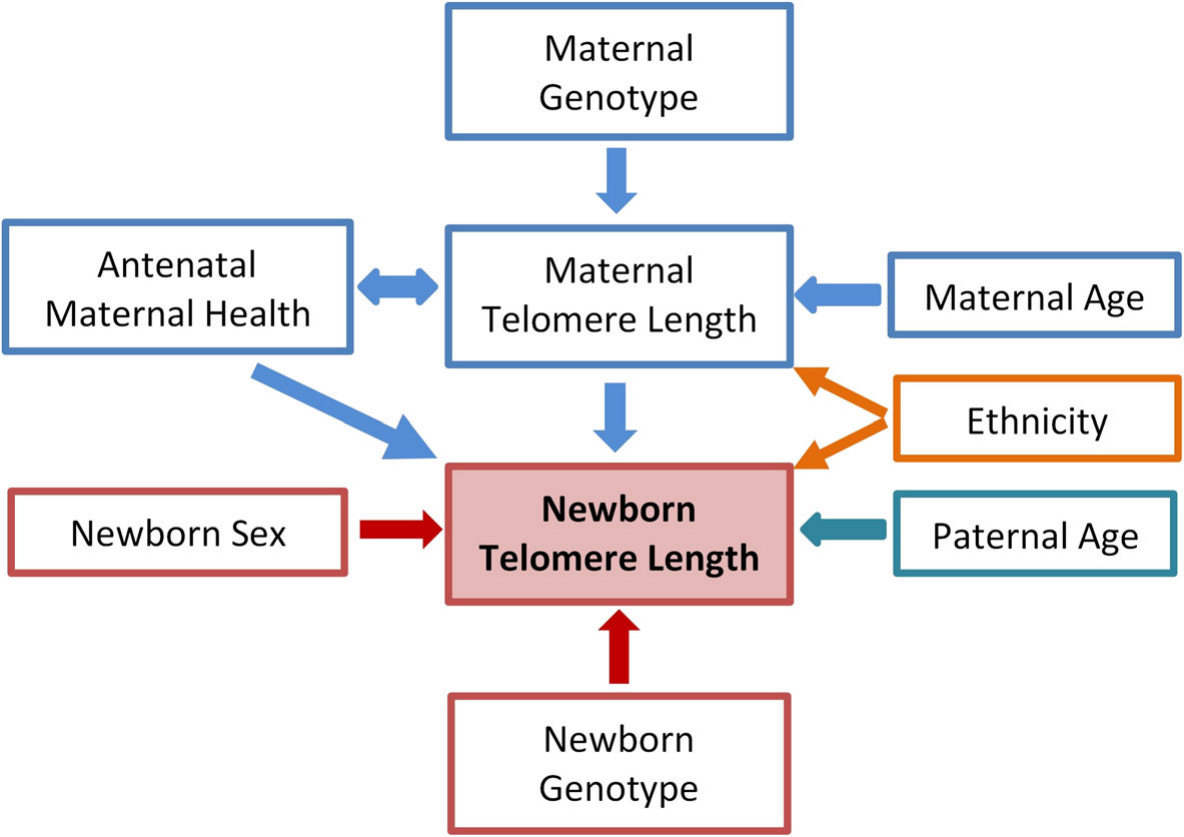
Schematic diagram summarizing the factors contributing to newborn TL variation

A schematic summary of all the heritable factors and the exposures contributing to the variation in newborn TL is shown in Fig. 3. In order to compare the contributions of individual factors in influencing the newborn TL variation, ten selected factors were studied by the model selection method using either all, male-only, or female-only subjects (the “Methods” section). Table 3 shows the results of the multivariate models that best explained newborn TL.

**Table 3:** The contributing factors that best explained newborn telomere length using all, male-only, and female-only subjects by model selection method. In each multivariate model, the factors were analyzed simultaneously for comparing their contributions to variation in newborn telomere length

Variable	All (*N* = 721)		Male (*N* = 378)		Female (*N* = 405)	
	*β* (95%CI)	*P* value	*β* (95%CI)	*P* value	*β* (95%CI)	*P* value
Sex						
Male	Ref	Ref	–	–	–	–
Female	0.44 (0.30,0.58)	7.42E−10***				
Ethnicity						
Chinese	Ref	Ref	Ref	Ref	Ref	Ref
Malay	− 0.17 (− 0.34, 0.00)	5.65E−02	− 0.10 (− 0.32, 0.13)	4.01E−01	− 0.33 (− 0.56, − 0.11)	4.16E−03*
Indian	− 0.22 (− 0.43, − 0.01)	3.73E−02*	− 0.20 (− 0.50, 0.09)	1.83E−01	− 0.21 (− 0.48,0.05)	1.19E−01
Newborn *LRRC34*- rs10936600 (0-AA 1-AT 2-TT)	− 0.20 (− 0.30, − 0.10)	1.70E−04**	− 0.23 (− 0.37, − 0.09)	1.53E−03*	− 0.15 (− 0.28,− 0.02)	2.86E−02*
Maternal telomere length (T/S)	0.12 (0.05, 0.19)	4.92E−04**	0.11 (0.01, 0.21)	3.16E−02*	0.16 (0.07, 0.25)	5.37E−04**
Paternal age (years)	0.13 (0.06, 0.20)	2.16E−04**	0.20 (0.10, 0.29)	3.43E−05**	–	–
Plasma fasting glucose (mmol/L)	− 0.08 (− 0.14, − 0.01)	2.42E−02*	− 0.15 (− 0.24, − 0.05)	3.91E−03*	–	–
Plasma IGFBP3 (ng/mL), log_10_	− 0.08 (− 0.15, − 0.02)	1.45E−02*	− 0.18 (− 0.27, − 0.09)	1.41E−04**	–	–
STAI Trait Score	–	–	–	–	− 0.13 (− 0.22, − 0.04)	4.12E−03*

*P < 0.05; **P < 0.001; ***P < 1.00E−06

Seven factors were found to best explain newborn TL variation in all subjects (*N* = 721). Among these, sex was the most significant factor (*β* = 0.44, *P* = 7.42E−10), followed by *LRRC34*-rs10936600 (*β* = − 0.20, *P* = 1.70E−04), paternal age (*β* = 0.13, *P* = 2.16E−04), maternal TL (*β* = 0.12, *P* = 4.92E−04), plasma IGFBP3 level (*β* = − 0.08, *P* = 1.45E−02), plasma fasting glucose concentration (*β* = − 0.08, *P* = 2.42E−02), and ethnicity (Indian vs Chinese: *β* = − 0.22, *P* = 3.73E−02). This model explained 12.4% of variance in newborn TL (Fig. S9A).

For male newborns, five key factors were identified in the best multivariate model (*N* = 378) that explained 12.6% of variance in TL (Fig. S9B). Paternal age was the most significant factor (*β* = 0.20, *P* = 3.43E−05), followed by plasma IGFBP3 level (*β* = − 0.18, *P* = 1.41E−04), *LRRC34*-rs10936600 (*β* = − 0.23, *P* = 1.53E−03), plasma fasting glucose concentration (*β* = − 0.15, *P* = 3.91E−03), and maternal TL (*β* = 0.11, *P* = 3.16E−02). Notably, paternal age, fasting glucose, and IGFBP3 levels were male TL-specific factors.

In female newborns, four factors were identified in the best multivariate model (*N* = 405) that explained 7.91% of variance in TL (Fig. S9C). Maternal TL showed the strongest association (*β* = 0.16, *P* = 5.37E−04). STAI trait score (*β* = − 0.13, *P* = 4.12E−03) ranked second, followed by ethnicity (Malay vs Chinese: *β* = − 0.33, *P* = 4.16E−03) and *LRRC34*-rs10936600 (*β* = − 0.15, *P* = 2.86E−02). STAI trait score was a female TL-specific factor.

### Sensitivity analysis

For sensitivity analysis, DNA storage time and sample collection month were further adjusted in the association studies with newborn and maternal TLs, respectively. For the best multivariate models of newborn TL (Table 3), these two factors did not show significant association after adding them in the models (Additional file 1: Table S7). For maternal TL, the association results were slightly affected (Additional file 1: Table S8). The associations of maternal TL with plasma PAI-1 and IGF2 levels lost significance in the supplementary model.

## Discussion

To the best of our knowledge, this is the first study to investigate newborn TL variation in umbilical cord tissue and report a comprehensive analysis of the effects of heritable factors, socioeconomic status, antenatal maternal health, and nutrition on this variation. It is known that TL is maximal at birth and decreases progressively with advancing age, and thus is considered a marker of biological aging [26]. In our study, we found relative average TL of newborns to be longer than the length of TL observed in their mothers (average maternal age, 31 years old). Maternal TL negatively correlated with maternal age ranging from 18 to 46 years. Newborn TL was not associated with gestational age but showed positive association with parental age. Comparison of the paternal and maternal age effects in the same regression model showed paternal age to have a dominant effect. This finding suggests that offsprings of older mothers could have longer TL in the analysis simply because the offsprings’ fathers were also older. Supporting this hypothesis, we indeed found a strong correlation between the parents’ age in our cohort. Previous studies [37–39] have reported increase in sperm TL with age as a potential reason for offsprings of older fathers to inherit longer telomeres. As oocytes are produced prenatally, while sperm are continually produced throughout life, it is believed that there is greater potential for TL plasticity with age in sperms than in oocytes. Effect of paternal age on newborn TL is intriguing as it potentiates a scenario of intergenerational genetic plasticity in which the DNA passed on to the offspring is systematically changed based upon the reproductive age of one’s father.

Genetic variants are additional factors known to have heritable effects on TL. The genome-wide association analysis in this study identified a LD block within the 3q26.2 region to be significantly associated with both maternal TL and newborn TL. *LRRC34*-rs10936600 was the top variant in this LD block. By using LDproxy and CHB population data [https://analysistools.nci.nih.gov/LDlink], we were able to extract the full list of genetic variants in a strong LD (*R*^2^ > 0.85) with rs10936600 (Additional file 1: Table S9). This analysis identified *TERC* variant rs2293607 (*R*^2^ = 0.98) to also be a member of this LD block. *TERC* encodes a long non-coding RNA found in eukaryotes that is a component of telomerase, the enzyme used to extend telomeres. The T allele of this variant (correlated to A allele in rs10936600) has been reported to be associated with an increase in *TERC* expression and TL [56]. Our finding is consistent with this reported trend as the dosage of A allele in rs10936600 is positively associated with TL (Fig. S4A). Further, the association results of previously reported genetic variants (*TERT*, *RTEL1*, *OBFC1*, *NAF1*, *ZNF208*, and *ACYP2*) are shown in Additional file 1: Table S10. They showed a weak association with TL in our study. Among them, *TERT*-rs2853677 (telomerase reverse transcriptase) showed the most significant association with maternal TL (*β* = 0.18, *P* = 1.85E−4).

For the previously reported factors associated with cord blood TL [25–36], we similarly found significant associations for sex, parental age, maternal TL, antenatal stress, maternal educational attainment, maternal smoking during pregnancy, and genomic region at 3q26.2, but the associations with maternal pre-pregnancy BMI, GDM status, hypertension, plasma folate, and vitamin D concentrations were not found in our study. As our pilot study (the “Methods” section) showed cord blood and cord tissue TLs were highly correlated (*R*^2^ = 0.64, Fig. S10), it is not surprising that we find similar associations with many factors. However, the discrepancy between our findings with the previous reports could be due to the sample size, population effects, and tissue-specific differences. In our study, we identified five new significant factors influencing newborn TL: ethnicity (Asian population), plasma fasting glucose concentration, plasma IGFBP3 level, plasma DGLA%, and vitamin B12 level (Table 2).

Sex-specific analysis identified distinct male vs female effects of factors influencing newborn TL. Interestingly, heritable factors such as maternal TL and newborn *LRRC34*-rs10936600 showed significant effects on both male and female newborn TLs with different effect sizes. *LRRC34*-rs10936600 showed a stronger effect on male newborns TL, while maternal TL had more pronounced effects on the female newborn TL. For other influencing factors, sex-specific effects were also observed. Female newborn TLs were more susceptible to the variation in maternal mental health (depression/anxiety) and vitamin B12 levels, while male newborn TLs were strongly affected by the variation of paternal age, maternal educational attainment, plasma fasting glucose concentration, plasma DGLA%, and IGFBP3 level. Although the negative impact of antenatal maternal distress on newborn TL has been widely reported, this study is the first to note that anxiety scores (STAI state and trait) have much stronger effects than depression scores (EPDS) on TL, and all scores exhibit female-specific effects. Low vitamin B12 level was linked to negative impacts on cognitive, motor, and growth outcomes for fetal development and was related to depression in mothers [57]. Interestingly, vitamin B12 level showed similar female-specific effects as did antenatal depression/anxiety. The effect of paternal age on offspring’s TL was linked to telomere elongation in sperm from older men as reported previously [39]. It is interesting to observe the male-specific effect of paternal age on newborn TL. For maternal educational attainment, the male-specific effect is consistent with a recent report that highlighted the male-specific effect of parental SES on newborn TL [58]. Impaired glucose metabolism and the related hormonal imbalance may generate physiological stress for the growing fetus [59] and may lead to TL attrition, especially for the male offspring. IGFBP3 is the main insulin-like growth factor transport protein in the bloodstream and plays an important role in senescence as an aging marker [60]. We observed a negative effect on male newborn TL. For all the TL-associated factors, we identified eight independent predictors of newborn TL (Table 3). These include sex, ethnicity, newborn *LRRC34*-rs10936600, maternal TL, paternal age, antenatal anxiety, plasma fasting glucose, and IGFBP3. The contributions of these factors influencing the variation in newborn TL were compared and ranked in three best multivariate models using all, female-only, and male-only subjects.

In the association study between maternal TL and antenatal maternal health, our results were similar to the findings from previous studies on TL associations with age [1], diabetes [6], 2-h post-load glucose concentration [7], educational attainment [17], and smoking status during pregnancy [5]. Although n-3 PUFA supplementation has been reported to affect TL [14], our study for the first time showed that higher plasma n-3 PUFA% and lower n-6:n-3 PUFA ratio are associated with longer maternal TLs. In addition, previous reports showed telomere shortening was associated with cardiovascular disease [12]. Elevated plasminogen activator inhibitor-1 (PAI-1) is a risk factor for thrombosis and atherosclerosis and associated with major adverse cardiovascular events (MACE) [61]. Notably, our study showed that shorter maternal TL was associated with higher plasma PAI-1 level. Finally, plasma IGF2 levels were positively associated with maternal TL. Higher circulating levels of IGF1, a related protein of IGF2, are known to be associated with longer TL in healthy subjects [62]. IGFs are known to play an essential role in the pathogenesis of several age-related diseases, including dementia, cardiovascular, and metabolic diseases; hence, their levels in circulation play a significant role in predicting healthspan and biological aging. The association of maternal TL with plasma PAI-1 and IGF2 levels should be interpreted with caution as their associations lost significance in the sensitivity analysis.

While comparing the factors influencing the newborn and maternal TLs, we found some consistent trends. For example, higher maternal educational attainment showed a positive association, and smoking during pregnancy showed a negative association with TL in both the mother and the offspring. The mediation analysis showed these two factors affected newborn TL through the mediating effect of maternal TL (Fig. S11). We also observed both mother and newborn TL to be associated with antenatal glycemia. As fasting and 2-h post-load glucose concentrations have different underlying etiologies and pathophysiologies, different effects on newborn/maternal TL are expected. It was noteworthy that maternal GDM or higher 2-h post-load glucose concentration was significantly associated with reduced TL only in mothers, while fasting glucose concentration had an adverse impact only on offsprings. Impaired glucose tolerance has been previously shown to associate with impaired telomerase activity [63] which may explain the shortening of TL in mothers. Although both impaired glucose tolerance and impaired fasting glucose can be harmful to the growing fetus [59], our findings showed that the latter had a more detrimental effect on fetal TL. Finally, we found that the association between antenatal depression/anxiety and TL was observed in the offspring but not in the mothers. This may be attributed to a high vulnerability of the fetus to stress during intrauterine development.

Our study has some limitations. First, the GUSTO cohort had a disproportionate number of participants within the Chinese, Malay, and Indian groups. Therefore, analyses of ethnic-specific effects of TL associated factors were limited by sample size. Second, due to the absence of paternal TL data in the cohort, we were unable to compare it with the effects of maternal TL on newborn TL. Finally, pre-pregnancy maternal weight, smoking status and alcohol consumption before/during pregnancy, and SES (maternal education and household income) were drawn from self-reported questionnaires. Bias may exist in the self-reported variables. Pre-pregnancy weight was assessed to be reliable as it highly correlated with the maternal weight at the first-trimester visit. In order to avoid underreported smoking, maternal smoking status during pregnancy was determined by the combination results of self-reported questionnaires and plasma cotinine level. As the DNA from maternal blood was extracted using two different methods (manual and automated), this technical variation could have impacted the TL measures. Hence, we adjusted the analysis models for this technical variation.

Telomeres serve as the biological timekeepers of cellular health, and hence, their attrition relative to chronological age is an indication of advanced biological aging, existence of a stressed environment, and potential risk to disease. Our study found antenatal maternal health to be a crucial determinant of TL programming in utero, which could potentially impact subsequent offspring health outcomes over the life span, including aging and longevity. Hence, improving antenatal health of mothers by targeting modifiable factors can help prevent in utero telomere attrition and enhance cellular longevity. As evident from the findings of this study, smoking during pregnancy, maternal nutrient insufficiency (i.e., vitamin B12), and cardiometabolic and mental health adversities are suboptimal conditions for fetal programming of telomere biology. In addition, our findings in sex-specific associations suggest different focus of antenatal care may be required for mothers carrying babies of different sex.

## Conclusions

We found evidence that the genetic variants at 3q26.2, paternal age, maternal TL, and antenatal maternal health have a significant impact on newborn TL. The comparative analysis of these factors identified the differences in the magnitude of their effects. Sex stratification analysis provided new insights into the factors explaining the male vs female TL variation. We also found that mother’s TL was significantly associated with her own metabolic health and nutrient status, which may have transgenerational effects on offspring TL. Our findings provide a comprehensive understanding of the heritable and environmental factors and their relative contributions to the initial setting of TL and programing of longevity in early life.

## Supplementary Information

**Additional file 1: Figure S1.** DNA quality analysis by agarose gel electrophoresis. **Figure S2.** Flowchart of sample selection and analysis steps. **Figure S3.** Trans-ethnic genome-wide association studies on telomere length. **Figure S4.** Boxplots of the top six genetic variants. **Figure S5.** Effect allele frequencies of the top six genetic variants. **Figure S6.** Heat map of pairwise Pearson correlation coefficients between clinical variables. **Figure S7.** Significant sex-specific effects of the selected factors on newborn telomere length. **Figure S8.** Association between maternal telomere length and antenatal maternal factors. **Figure S9.** The variance percentage explained by each factor. **Figure S10.** Scatter plot of average relative telomere length of cord blood and cord tissue. **Figure S11.** Mediation analysis of maternal telomere length. **Table S1.** Intra-class correlation coefficient of intra-assay and inter-assay for telomere length measurements. **Table S2.** Comparison of the basic characteristics of 950 subjects and the full cohort. **Table S3.** Clinical characteristics of maternal-offspring subjects in this study and linear regression results for newborn TL. **Table S4.** The association of SNPs at 3q26.2 in the GWAS results of newborn and maternal telomere lengths and the meta-analysis results. **Table S5.** Pairwise Linkage Disequilibrium measures between the top six genetic variants. **Table S6.** Linear regression results between maternal telomere length and antenatal maternal factors. **Table S7.** The results of sensitivity analysis after adding DNA storage time and sample collection month in the best multivariate models of newborn telomere length. **Table S8.** The results of sensitivity analysis after further adjustment for DNA storage time and sample collection month in the association studies between maternal telomere length and antenatal maternal factors. **Table S9.** The genetic variants in a strong Linkage Disequilibrium with rs10936600. **Table S10.** The association of candidate genes in the GWAS results of newborn and maternal telomere lengths and the meta-analysis results.

## Data Availability

Data are not publicly available due to ethical restrictions but can be obtained from the authors upon reasonable request and subject to appropriate approvals, including from the GUSTO cohort’s Executive Committee.

## Abbreviations

AA: Arachidonic acid
APGAR: A score is determined by evaluating the newborn baby on five simple criteria on a scale from 0 to 2
DGLA: Dihomo-gamma-linolenic acid
DHA: Docosahexaenoic acid
DOHaD: Developmental origins of health and disease
DPA: Docosapentaenoic acid
EPA: Eicosapentaenoic acid
EPDS: Edinburgh Postnatal Depression Scale
GA: Gestational age
GDM: Gestational diabetes mellitus
GUSTO: Growing Up in Singapore Towards healthy Outcomes
GWAS: Genome-wide association study
GWG: Gestational weight gain
IGF: Insulin-like growth factor
IGFBP: Insulin-like growth factor-binding protein
LA: Linoleic acid
LD: Linkage disequilibrium
MUFA: Mono-unsaturated fatty acid
PAI-1: Plasminogen activator inhibitor-1
ppBMI: Pre-pregnancy body mass index
PUFA: Poly-unsaturated fatty acid
SD: Standard deviation
SFA: Saturated fatty acid
SNP: Single-nucleotide polymorphism
STAI: State-Trait Anxiety Inventory
TL: Telomere length
