# Supplementary material for: Variability in newborn telomere length is explained by inheritance and intrauterine environment
Source: BMC Med. 2022 Jan 25;20:20. doi: 10.1186/s12916-021-02217-9 (PMC8787951; doi:10.1186/s12916-021-02217-9)
Supplement: Supplementary file 1 — Additional file 1: Figure S1. DNA quality analysis by agarose gel electrophoresis. Figure S2. Flowchart of sample selection and analysis steps. Figure S3. Trans-ethnic genome-wide association studies on telomere length. Figure S4. Boxplots of the top six genetic variants. Figure S5. Effect allele frequencies of the top six genetic variants. Figure S6. Heat map of pairwise Pearson correlation coefficients between clinical variables. Figure S7. Significant sex-specific effects of the selected factors on newborn telomere length. Figure S8. Association between maternal telomere length and antenatal maternal factors. Figure S9. The variance percentage explained by each factor. Figure S10. Scatter plot of average relative telomere length of cord blood and cord tissue. Figure S11. Mediation analysis of maternal telomere length. Table S1. Intra-class correlation coefficient of intra-assay and inter-assay for telomere length measurements. Table S2. Comparison of the basic characteristics of 950 subjects and the full cohort. Table S3. Clinical characteristics of maternal-offspring subjects in this study and linear regression results for newborn TL. Table S4. The association of SNPs at 3q26.2 in the GWAS results of newborn and maternal telomere lengths and the meta-analysis results. Table S5. Pairwise Linkage Disequilibrium measures between the top six genetic variants. Table S6. Linear regression results between maternal telomere length and antenatal maternal factors. Table S7. The results of sensitivity analysis after adding DNA storage time and sample collection month in the best multivariate models of newborn telomere length. Table S8. The results of sensitivity analysis after further adjustment for DNA storage time and sample collection month in the association studies between maternal telomere length and antenatal maternal factors. Table S9. The genetic variants in a strong Linkage Disequilibrium with rs10936600. Table S10. The association of candidate genes in the GWAS [file 12916_2021_2217_MOESM1_ESM.docx]

**Supplementary Information**

**Variability in newborn telomere length is explained by inheritance and intrauterine environment**

Li Chen, Karen Tan Mei Ling, Min Gong, Mary F.F. Chong, Kok Hian Tan, Yap Seng Chong, Michael J. Meaney, Peter D. Gluckman, Johan G. Eriksson and Neerja Karnani

**Additional File 1: Fig. S1 – S11 and Table S1 – S10**

**Fig. S1.** DNA quality analysis by agarose gel electrophoresis

**Fig. S2.** Flowchart of sample selection and analysis steps

**Fig. S3.** Trans-ethnic genome-wide association studies on telomere length

**Fig. S4.** Boxplots of the top six genetic variants

**Fig. S5.** Effect allele frequencies of the top six genetic variants

**Fig. S6.** Heat map of pairwise Pearson correlation coefficients between clinical variables

**Fig. S7.** Significant sex-specific effects of the selected factors on newborn telomere length

**Fig. S8.** Association between maternal telomere length and antenatal maternal factors.

**Fig. S9.** The variance percentage explained by each factor

**Fig. S10.** Scatter plot of average relative telomere length of cord blood and cord tissue

**Fig. S11.** Mediation analysis of maternal telomere length

**Table S1**. Intra-class correlation coefficient of intra-assay and inter-assay for telomere length measurements

**Table S2.** Comparison of the basic characteristics of 950 subjects and the full cohort

**Table S3.** Clinical characteristics of maternal-offspring subjects in this study and linear regression results for newborn TL

**Table S4.** The association of SNPs at 3q26.2 in the GWAS results of newborn and maternal telomere lengths and the meta-analysis results

**Table S5.** Pairwise Linkage Disequilibrium measures between the top six genetic variants

**Table S6.** Linear regression results between maternal telomere length and antenatal maternal factors

**Table S7.** The results of sensitivity analysis after adding DNA storage time and sample collection month in the best multivariate models of newborn telomere length

**Table S8.** The results of sensitivity analysis after further adjustment for DNA storage time and sample collection month in the association studies between maternal telomere length and antenatal maternal factors

**Table S9.** The genetic variants in a strong Linkage Disequilibrium with rs10936600

**Table S10.** The association of candidate genes in the GWAS results of newborn and maternal telomere lengths and the meta-analysis results

**1kb Ladder**

**1 2 3 4 5 6 7 8 9 10 11 12 13 14 15 16 17 18 19 20 21 22 23 24**

A


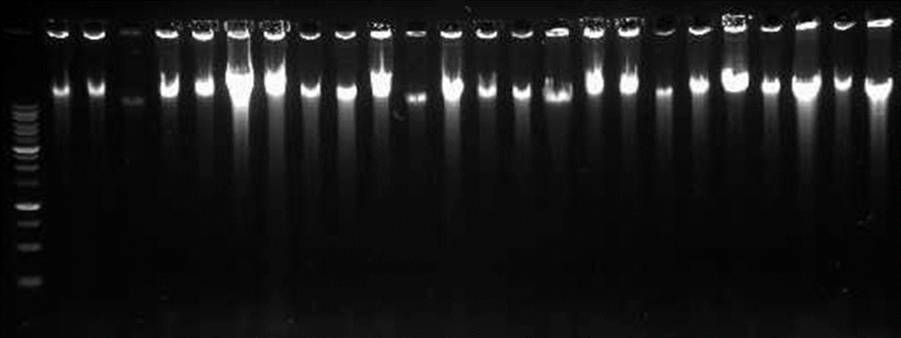


**10kb**

**1kb Ladder**

B

**1 2 3 4 5 6 7 8 9 10 11 12 13 14 15 16 17 18 19 20 21 22 23 24 25**


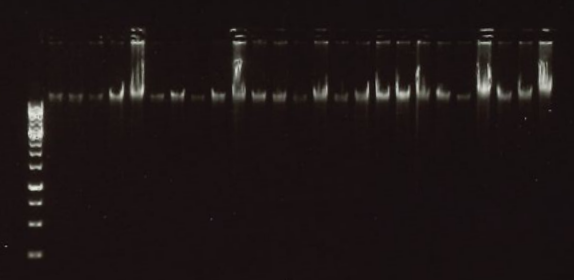


**10kb**

**Fig. S1.** DNA quality analysis by agarose gel electrophoresis. Examples of gel images for **(A)** cord tissue DNA and **(B)** maternal buffy coat DNA

**GUSTO cohort**

**(1247 Subjects)**

1. APGAR≥9 and singleton

2. Available sex, ethnicity, maternal age, gestational

age and newborn genotype data

**950 subjects with newborn TL**

**Step 1: Sex, ethnicity and age effects on newborn TL**

**Step 2: Inheritance of TL (maternal TL; GWAS of newborn & maternal TL)**

**Step 3: Association study between newborn TL and antenatal maternal factors**

**Step 4: Association study between maternal TL (N=892) and antenatal maternal factors**

**Step 5: Sex stratification analysis on newborn TL**

**Step 6: Determinants of newborn TL**

**Fig. S2.** Flowchart of sample selection and analysis steps in this study


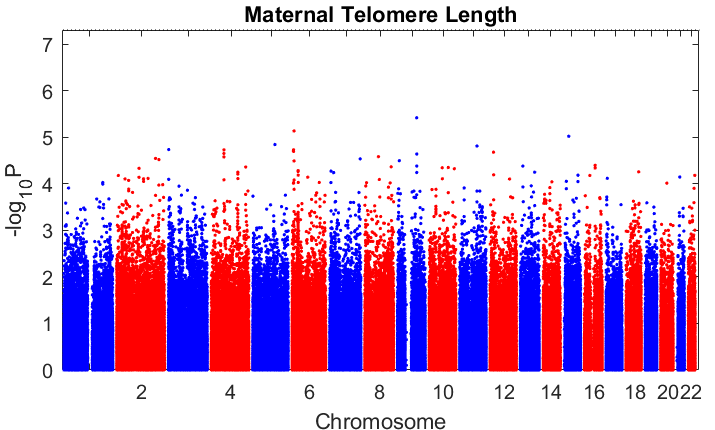

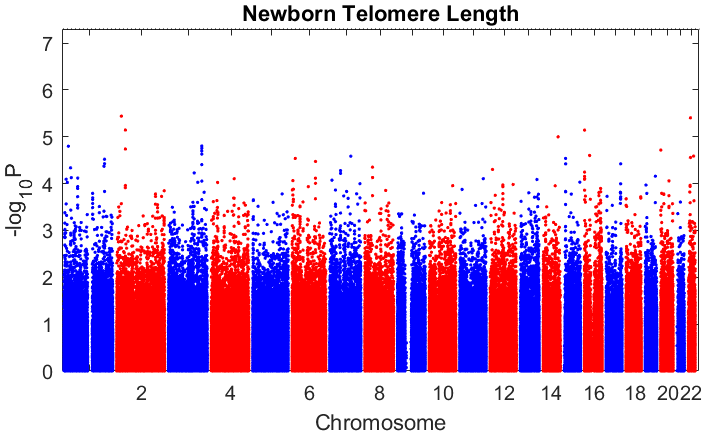


C

B

A


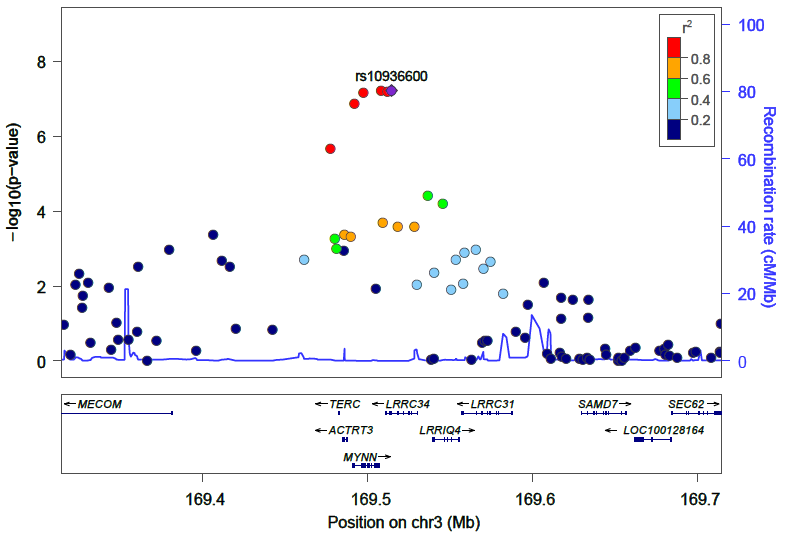

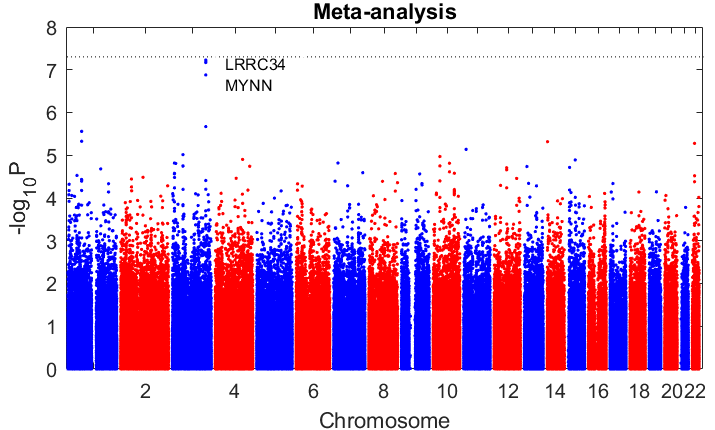


D

**Fig. S3.** Trans-ethnic genome-wide association studies (GWAS) on telomere length. **(A)** Manhattan plot of the GWAS results of newborn telomere length. **(B)** Manhattan plot of the GWAS results of maternal telomere length. **(C)** Manhattan plot of the meta-analysis results of two GWAS studies of newborn and maternal telomere lengths. Dotted line – genome-wide significance cut-off (P=5.00E-08). **(D)** The locus zoom plot for the top six genetic variants in the meta-analysis results using hg19/1000 Genomes Nov 2014 ASN.

B

A


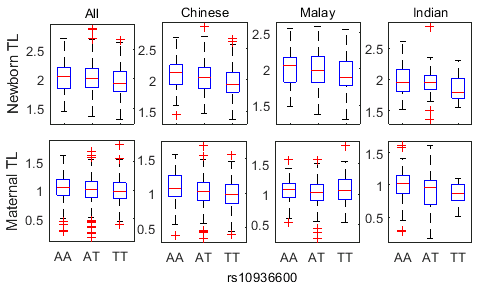

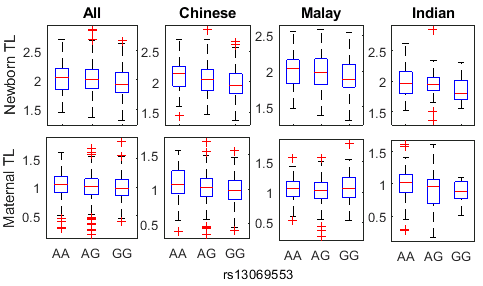


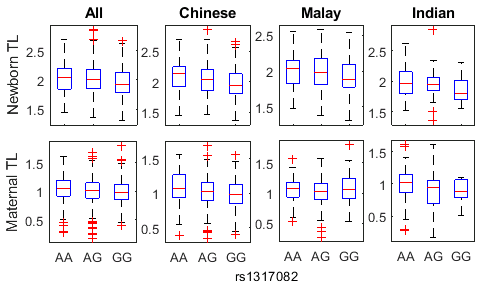


C

D


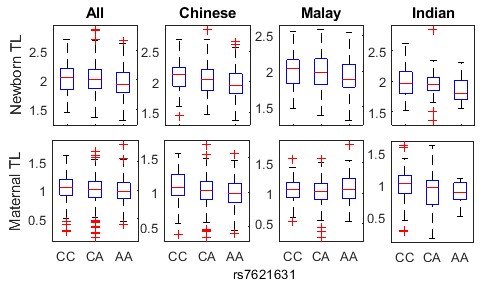


F

E


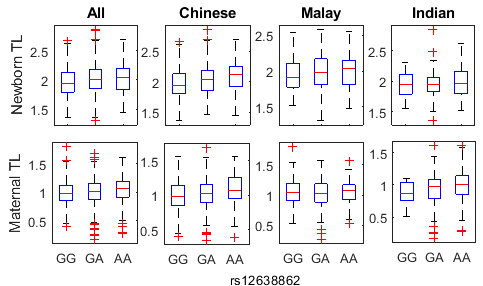


**Fig. S4.** Boxplots of the top six genetic variants in the meta-analysis results using all, Chinese, Malay and Indian subjects. **(A)** rs10936600 (*LRRC34*) **(B)** rs13069553 (767 bp downstream of *MYNN*) **(C)** rs7621631 (*LRRC34*) **(D)** rs1317082 (*MYNN*) **(E)** rs10936599 (*MYNN*) **(F)** rs12638862 (4891 bp downstream of *TERC*)

A

B

**Fig. S5.** Effect allele frequencies of the top six genetic variants in three ethnicities (reference/effect allele). **(A)** Newborn Genotype **(B)** Maternal Genotype

**Fig. S6.** Heat map of pairwise Pearson correlation coefficients between clinical variables (continuous measures in Table S3)

**Fig. S7.** Significant sex-specific effects of the selected factors on newborn telomere length (P values after adjustment for ethnicity) **(A)** Stronger effect on male newborns, i.e. scatter plots of paternal age, plasma fasting glucose concentration and plasma IGFBP3 level (log-transformed). **(B)** Stronger effect on female newborns, i.e. scatter plots of maternal TL, STAI trait score and plasma vitamin B12 level (log-transformed). Related to Table 2.

**Fig. S8.** Association between maternal telomere length and antenatal maternal factors. Scatter/box plots for nine significant factors: GDM status, education (1: Secondary and below; 2: Post-secondary; 3: University), smoking status during pregnancy, plasma 2-h post-load glucose concentration, plasma folate level (log-transformed), plasma IGF2 level (log-transformed), plasma total n-3 PUFA%, plasma n-6:n-3 PUFA ratio and plasma PAI-1 level (log-transformed). P values are obtained after adjustment for maternal age, ethnicity and DNA extraction method. Related to Table S6.


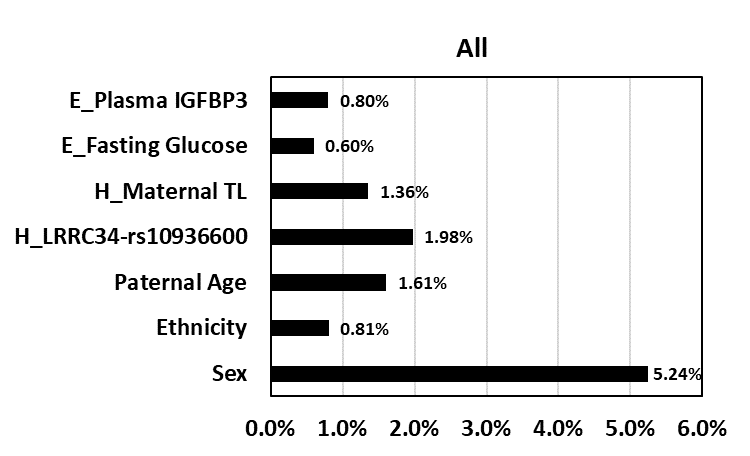


**A**


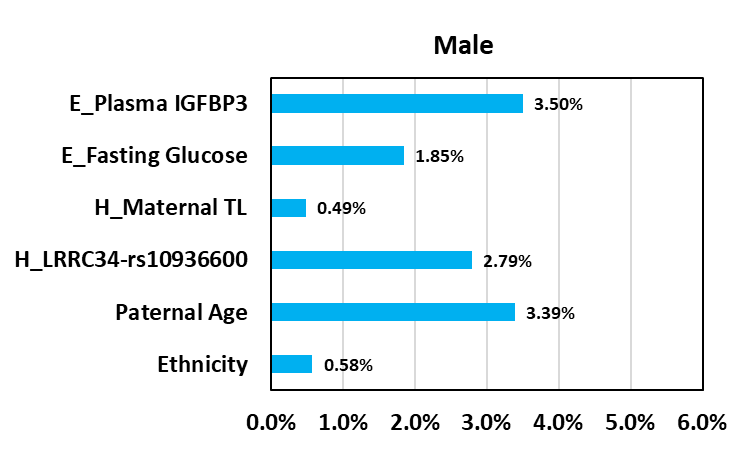


**C**

**B**


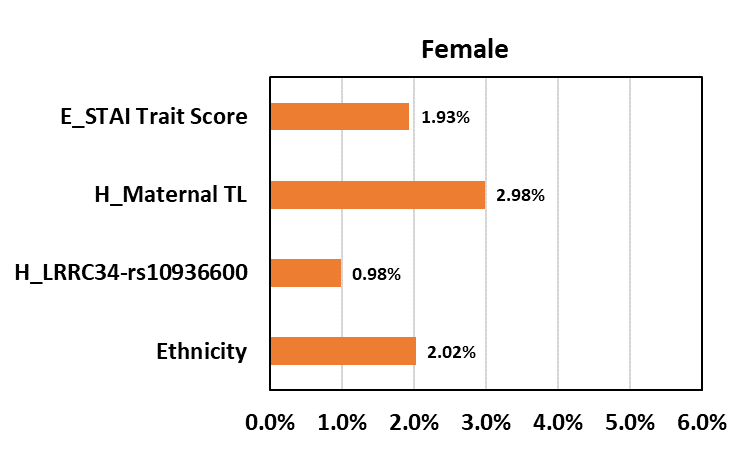


**Fig. S9.** The variance percentage explained by each factor in the best multivariate models of newborn telomere length using (**A**) all, (**B**) male only and (**C**) female only subjects in Table 3. H-heritable factor and E-environmental factor.


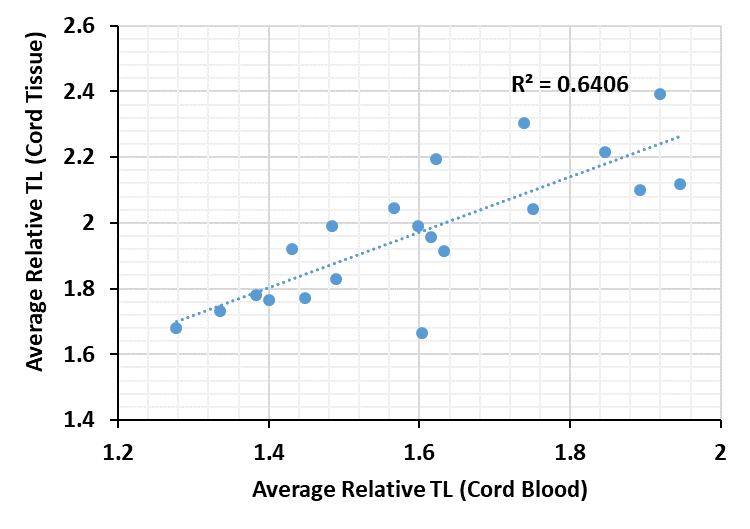


**Fig. S10.** Scatter plot of average relative telomere length of cord blood and cord tissue in the pilot study (20 subjects)

**B**

**A**


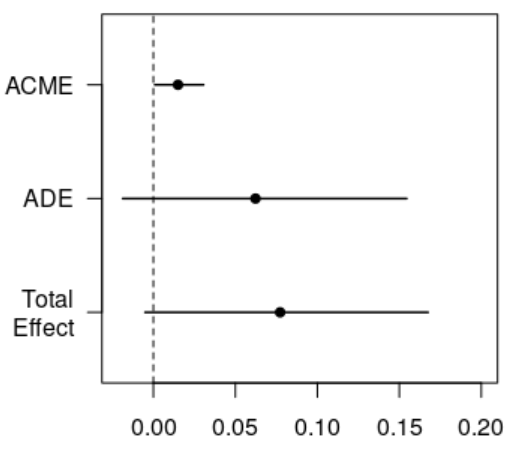

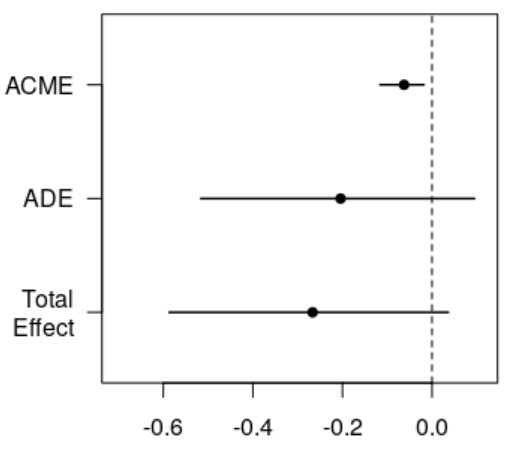


Effect Size

Effect Size

**Fig. S11.** Mediation analysis of maternal telomere length for the effects of **(A)** maternal educational attainment (N=880; P_ACME_=0.028) and **(B)** smoking status during pregnancy (N=781; P_ACME_=0.004) on newborn telomere length after adjustment for sex, ethnicity, maternal age, DNA extraction method of maternal blood samples. ACME: average causal mediation effect and ADE: average direct effect (mediation R package, nonparametric bootstrap confidence intervals with the percentile method).

**Table S1**. Intra-class correlation coefficient (ICC) of intra-assay and inter-assay for telomere length measurements in cord tissue (9 assay plates) and maternal DNA samples (10 assay plates)

| Cord Tissue DNA Samples | | | | Maternal DNA Samples | | | |
| --- | --- | --- | --- | --- | --- | --- | --- |
| Assay | | ICC | 95% CI | Assay | | ICC | 95%CI |
| Inter-assay | | 0.924 | [0.913, 0.934] | Inter-assay | | 0.978 | [0.975, 0.980] |
| Intra-assay | 1 | 0.958 | [0.937, 0.971] | Intra-assay | 1 | 0.992 | [0.989, 0.995] |
|  | 2 | 0.920 | [0.881, 0.945] |  | 2 | 0.982 | [0.973, 0.988] |
|  | 3 | 0.920 | [0.885, 0.946] |  | 3 | 0.984 | [0.977, 0.989] |
|  | 4 | 0.909 | [0.867, 0.937] |  | 4 | 0.973 | [0.959, 0.982] |
|  | 5 | 0.881 | [0.820, 0.918] |  | 5 | 0.976 | [0.964, 0.984] |
|  | 6 | 0.932 | [0.898, 0.954] |  | 6 | 0.949 | [0.924, 0.965] |
|  | 7 | 0.883 | [0.830, 0.920] |  | 7 | 0.962 | [0.945, 0.975] |
|  | 8 | 0.929 | [0.894, 0.951] |  | 8 | 0.927 | [0.892, 0.950] |
|  | 9 | 0.947 | [0.915, 0.966] |  | 9 | 0.909 | [0.868, 0.939] |
|  | --- | --- | --- |  | 10 | 0.955 | [0.931, 0.970] |

# **Table S2**. Comparison of the basic characteristics of 950 subjects and the full cohort

| Variables | This Study  (N=950) | Cohort  (N=1177*) | P Value |
| --- | --- | --- | --- |
|  | Mean(SD) / N(%) | Mean(SD) / N(%) |  |
| Ethnicity  Chinese  Malay  Indian | 555 (58.4%)  234 (24.6%)  161 (17.0%) | 662 (56.2%)  299 (25.4%)  216 (18.4%) | 5.64E-01 |
| Sex  Male  Female | 500 (52.6%)  450 (47.4%) | 619 (52.6%)  558 (47.4%) | 9.85E-01 |
| Gestational Age (weeks) | 38.81(1.34) | 38.70(1.59) | 1.06E-01 |
| Birth Weight (kg) | 3.11(0.43) | 3.08(0.47) | 1.09E-01 |
| Maternal Age (years) | 30.82(5.08) | 30.65(5.09) | 4.32E-01 |

*- the total number of live singleton births in the GUSTO cohort

**Table S3.** Clinical characteristics of maternal-offspring subjects in this study and linear regression results for newborn TL

| Category | Variables | N | Mean (SD) / % | Univariate Analysis | | Multivariate Analysis  (Adjusted for gender and ethnicity) | |
| --- | --- | --- | --- | --- | --- | --- | --- |
|  |  |  |  | β (95%CI) | P Value | β (95%CI) | P Value |
| TL | Newborn Telomere Length (T/S) | 950 | 2.00 (0.25) | --- | --- | --- | --- |
| Newborn  Sex | Male  Female | 500  450 | 52.6%  47.4% | Ref.  0.40(0.28,0.53) | Ref.  **4.45E-10***** | --- | **---** |
| Ethnicity | Chinese  Malay  Indian | 555  234  161 | 58.4%  24.6%  17.0% | Ref.  -0.21(-0.36,-0.06)  -0.21(-0.39,-0.04) | Ref.  **6.63E-03***  **1.67E-02*** | --- | **---** |
| Inheritance | Maternal Telomere Length^a^ (T/S) | 892 | 1.02 (0.23) | 0.15(0.08,0.21) | **9.47E-06**** | 0.14(0.08,0.20) | **1.99E-05**** |
| Birth Outcomes | Birth Weight (kg)  Gestational Age (weeks) | 950  950 | 3.11 (0.43)  38.81 (1.34) | 0.02(-0.04,0.08)  0.02(-0.04,0.09) | 5.40E-01  4.96E-01 | 0.05(-0.02,0.11)  0.01(-0.05,0.07) | 1.58E-01  7.47E-01 |
| Parental Age | Maternal Age (years) | 950 | 30.82 (5.08) | 0.09(0.02,0.15) | **8.69E-03*** | 0.07(0.01,0.14) | **2.78E-02*** |
|  | Paternal Age (years) | 805 | 34.09 (5.84) | 0.11(0.04,0.18) | **1.25E-03*** | 0.10(0.03,0.17) | **3.73E-03*** |
| Maternal Mental Health^a^ | EPDS Score | 918 | 7.53 (4.43) | -0.06(-0.12,0.01) | 8.45E-02 | -0.05(-0.11,0.02) | 1.40E-01 |
|  | STAI State Score | 895 | 34.58 (9.76) | -0.08(-0.14,-0.01) | **2.00E-02*** | -0.07(-0.14,-0.01) | **2.93E-02*** |
|  | STAI Trait Score | 891 | 36.70 (8.90) | -0.09(-0.16,-0.03) | **4.86E-03*** | -0.09(-0.16,-0.03) | **3.61E-03*** |
| Maternal Adiposity | Pre-pregnancy BMI (kg/m^2^) | 871 | 22.73 (4.32) | -0.07(-0.14,-0.01) | **2.78E-02*** | -0.05(-0.12,0.02) | 1.67E-01 |
|  | Gestation Weight Gain^a^ (kg) | 869 | 8.60 (4.49) | -0.01(-0.08,0.06) | 7.50E-01 | 0.01(-0.06,0.07) | 8.38E-01 |
|  | Height (cm) | 930 | 158.17 (5.57) | 0.06(0.00,0.13) | 5.54E-02 | 0.05(-0.01,0.11) | 1.13E-01 |
| Maternal Blood Pressure^a^ | Systolic BP (mmHg) | 800 | 113.22 (12.56) | -0.07(-0.13,0.01) | 6.09E-02 | -0.05(-0.12,0.01) | 1.12E-01 |
|  | Diastolic BP (mmHg) | 800 | 64.64 (8.49) | -0.02(-0.08,0.05) | 6.55E-01 | 0.00(-0.07,0.07) | 9.56E-01 |
| Maternal Plasma Glucose Concentration^a^ | Fasting Glucose (mmol/L) | 905 | 4.34 (0.43) | -0.07(-0.13,0.00) | **3.74E-02*** | -0.08(-0.14,-0.01) | **1.60E-02*** |
|  | 2-h Post-load Glucose (mmol/L) | 905 | 6.53 (1.45) | -0.01(-0.08,0.06) | 7.73E-01 | -0.02(-0.08,0.05) | 5.67E-01 |
|  | Gestational Diabetes Mellitus  0: Non-GDM  1: GDM | 739  166 | 81.7%  18.3% | -0.03(-0.20,0.14) | 7.20E-01 | -0.04(-0.20,0.13) | 6.56E-01 |
| Maternal Plasma Fatty Acid^a^ | Total SFA% | 832 | 45.85 (3.38) | -0.02(-0.09,0.04) | 4.84E-01 | -0.01(-0.08,0.06) | 7.30E-01 |
|  | Total MUFA% | 832 | 13.60 (2.28) | -0.03(-0.1,0.04) | 4.10E-01 | -0.01(-0.08,0.05) | 6.77E-01 |
|  | Total n-6 PUFA% | 832 | 34.17 (3.33) | 0.02(-0.05,0.09) | 5.55E-01 | 0.01(-0.06,0.08) | 7.72E-01 |
|  | LA% | 832 | 21.73 (3.42) | 0.02(-0.05,0.09) | 5.37E-01 | 0.01(-0.05,0.08) | 7.01E-01 |
|  | DGLA% | 831 | 3.92 (1.27) | -0.03(-0.1,0.04) | 3.66E-01 | -0.03(-0.09,0.04) | 4.65E-01 |
|  | AA% | 832 | 7.89 (1.68) | 0.01(-0.06,0.07) | 8.55E-01 | 0.00(-0.07,0.07) | 9.71E-01 |
|  | Total n-3 PUFA% | 832 | 6.38 (1.84) | 0.04(-0.03,0.11) | 2.16E-01 | 0.02(-0.05,0.09) | 5.41E-01 |
|  | DHA% | 832 | 4.72 (1.42) | 0.03(-0.04,0.10) | 4.04E-01 | 0.01(-0.06,0.08) | 7.31E-01 |
|  | DPA% | 832 | 0.60 (0.20) | 0.02(-0.05,0.09) | 5.09E-01 | 0.01(-0.06,0.08) | 8.35E-01 |
|  | EPA% | 832 | 0.69 (0.56) | 0.04(-0.03,0.11) | 2.19E-01 | 0.03(-0.04,0.09) | 4.56E-01 |
|  | n-6:n-3 PUFA Ratio | 832 | 5.85 (1.95) | -0.02(-0.09,0.04) | 4.94E-01 | -0.01(-0.08,0.06) | 7.31E-01 |
| Maternal Plasma Vitamins and Metabolites^a^ | Vitamin B6 (nmol/L), log_10_ | 825 | 1.85 (0.36) | 0.06(-0.01,0.12) | 1.14E-01 | 0.06(-0.01,0.13) | 8.01E-02 |
|  | Vitamin B12 (pg/ml), log_10_ | 834 | 2.45 (0.15) | 0.08(0.01,0.15) | **1.91E-02*** | 0.08(0.01,0.14) | **2.87E-02*** |
|  | Folate (ng/ml), log_10_ | 834 | 1.15 (0.29) | 0.04(-0.03,0.11) | 2.35E-01 | 0.02(-0.05,0.09) | 6.28E-01 |
|  | Vitamin D_3_ (nmol/L) | 794 | 82.12 (27.38) | 0.08(0.01,0.15) | **2.00E-02*** | 0.06(-0.02,0.13) | 1.23E-01 |
|  | Betaine (µmol/L), log_10_ | 825 | 1.11 (0.09) | -0.03(-0.10,0.03) | 3.31E-01 | -0.03(-0.10,0.04) | 3.91E-01 |
|  | Choline (µmol/L), log_10_ | 825 | 0.96 (0.08) | -0.02(-0.09,0.05) | 5.86E-01 | -0.03(-0.09,0.04) | 4.50E-01 |
|  | Homocysteine (µmol/L), log_10_ | 825 | 0.69 (0.09) | 0.00(-0.07,0.06) | 8.89E-01 | 0.01(-0.06,0.07) | 8.50E-01 |
| Maternal Plasma Protein Biomarkers^a^ | Adiponectin (pg/ml), log_10_ | 939 | 7.16 (0.27) | -0.01(-0.07,0.05) | 7.70E-01 | -0.02(-0.08,0.04) | 5.28E-01 |
|  | Leptin (pg/ml), log_10_ | 922 | 4.18 (0.39) | -0.02(-0.09,0.04) | 4.73E-01 | 0.00(-0.07,0.06) | 9.49E-01 |
|  | C-Reactive Protein (ng/ml), log_10_ | 938 | 4.75 (0.48) | -0.05(-0.12,0.01) | 1.15E-01 | -0.04(-0.10,0.03) | 2.87E-01 |
|  | PAI-1 (pg/ml), log_10_ | 932 | 4.20 (0.22) | -0.04(-0.1,0.03) | 2.53E-01 | -0.03(-0.09,0.03) | 3.37E-01 |
|  | IGF1 (pg/ml), log_10_ | 939 | 4.72 (0.16) | -0.03(-0.09,0.04) | 4.14E-01 | -0.02(-0.08,0.04) | 5.07E-01 |
|  | IGF2 (pg/ml), log_10_ | 848 | 5.40 (0.24) | 0.04(-0.03,0.11) | 2.38E-01 | 0.01(-0.05,0.08) | 6.65E-01 |
|  | IGFBP1 (ng/ml), log_10_ | 939 | 1.15 (0.26) | 0.05(-0.02,0.11) | 1.39E-01 | 0.04(-0.02,0.11) | 1.69E-01 |
|  | IGFBP3 (ng/ml), log_10_ | 938 | 2.74 (0.18) | -0.09(-0.16,-0.03) | **4.35E-03*** | -0.08(-0.15,-0.02) | **7.62E-03*** |
|  | IGFBP4 (ng/ml), log_10_ | 917 | 1.21 (0.27) | 0.00(-0.06,0.07) | 9.11E-01 | 0.01(-0.06,0.07) | 8.37E-01 |
|  | IGFBP7 (ng/ml), log_10_ | 939 | 1.73 (0.12) | -0.02(-0.08,0.04) | 5.17E-01 | -0.03(-0.10,0.03) | 2.96E-01 |
| Socio-economic  Status  (SES) | Maternal Education  1: Secondary and below  2: Post-secondary  3: University | 283  336  319 | 30.2%  35.8%  34.0% | 0.10(0.02,0.18) | **1.70E-02*** | 0.07(-0.01,0.16) | 7.79E-02 |
|  | Household Income  1: ≤S$1999  2: S$2000-5999  3:≥S$6000 | 131  492  264 | 14.8%  55.5%  29.8% | 0.12(0.02,0.22) | **1.72E-02*** | 0.10(-0.01,0.20) | 7.10E-02 |
| Maternal Smoking Status | Before pregnancy  0: No  1: Yes | 816  121 | 87.1%  12.9% | -0.21(-0.40,-0.02) | **3.15E-02*** | -0.16(-0.35,0.03) | 9.59E-02 |
|  | During pregnancy  0: No  1: Yes | 788  39 | 95.3%  4.7% | -0.40(-0.72,-0.08) | **1.45E-02*** | -0.35(-0.67,-0.03) | **3.06E-02*** |
| Maternal Alcohol Consumption | Before pregnancy  0: No  1: Yes | 600  339 | 63.9%  36.1% | 0.02(-0.12,0.15) | 8.14E-01 | -0.07(-0.21,0.07) | 3.27E-01 |
|  | During pregnancy  0: No  1: Yes | 899  21 | 97.7%  2.3% | 0.50(0.06,0.93) | **2.47E-02*** | 0.35(-0.08,0.77) | 1.09E-01 |
| Parity | 0: Primiparous  1: Multiparous | 430  520 | 45.3%  54.7% | -0.06(-0.18,0.07) | 3.88E-01 | -0.02(-0.15,0.11) | 7.50E-01 |

P<0.05 *; P<0.001**; P<1.00E-06***; Underscore: borderline P value; ^a^: measured at 26-28 weeks’ pregnancy; β: effect size; CI: confidence interval; log_10_: those variables with skewed distribution are log10-transformed for the calculation of mean and standard deviation and for linear regression analysis; EPDS: Edinburgh Postnatal Depression Scale; STAI: The State-Trait Anxiety Inventory; SFA: Saturated Fatty Acid; MUFA: Mono-Unsaturated Fatty Acid; PUFA: Poly-Unsaturated Fatty Acid; LA: Linoleic Acid; DGLA: Dihomo-Gamma-Linolenic Acid; AA: Arachidonic Acid; DHA: Docosahexaenoic Acid; DPA: Docosapentaenoic Acid; EPA: Eicosapentaenoic Acid; IGF: Insulin-like Growth Factor; IGFBP: Insulin-like Growth Factor Binding Protein; PAI-1: Plasminogen Activator Inhibitor-1

**Table S4.** The association of SNPs at 3q26.2 in the GWAS results of newborn and maternal telomere lengths and the meta-analysis results. The SNPs with P<1.00E-05 found by meta-analysis are highlighted as grey. Significant P values (P<0.05) are highlighted as bold. Genetic variants are ordered by their genomic positions.

| **rsID** | **Newborn TL** | | **Maternal TL** | | **Meta-analysis** | | **Annotation** | | | |
| --- | --- | --- | --- | --- | --- | --- | --- | --- | --- | --- |
|  | **β(SE)** | **P Value** | **β(SE)** | **P Value** | **β(SE)** | **P Value** | **Chr** | **Position^#^** | **EA** | **Features** |
| rs10936590 | -0.12(0.05) | **1.40E-02** | -0.12(0.05) | **1.18E-02** | -0.12(0.03) | **4.26E-04** | 3 | 169406484 | A | region is 75913 bp downstream of TERC (-) |
| rs1421018 | -0.09(0.05) | **4.82E-02** | -0.12(0.05) | **1.73E-02** | -0.1(0.03) | **2.11E-03** | 3 | 169411792 | G | region is 70605 bp downstream of TERC (-) |
| rs16854319 | -0.1(0.05) | **3.85E-02** | -0.11(0.05) | **3.50E-02** | -0.1(0.03) | **3.10E-03** | 3 | 169416569 | C | region is 65828 bp downstream of TERC (-) |
| rs1997397 | -0.07(0.05) | 1.36E-01 | -0.03(0.05) | 5.59E-01 | -0.05(0.03) | 1.39E-01 | 3 | 169420117 | G | region is 62280 bp downstream of TERC (-) |
| rs9880534 | -0.05(0.05) | 2.38E-01 | -0.04(0.05) | 3.94E-01 | -0.05(0.03) | 1.50E-01 | 3 | 169442363 | G | region is 40034 bp downstream of TERC (-) |
| rs16854335 | -0.1(0.05) | **2.91E-02** | -0.11(0.05) | **2.79E-02** | -0.1(0.03) | **1.92E-03** | 3 | 169461571 | A | region is 20826 bp downstream of TERC (-) |
| rs12638862 | 0.16(0.05) | **5.86E-04** | 0.15(0.05) | **1.18E-03** | 0.16(0.03) | **2.14E-06** | 3 | 169477506 | A | region is 4891 bp downstream of TERC (-) |
| rs12630450 | 0.15(0.05) | **1.75E-03** | 0.09(0.05) | 8.25E-02 | 0.12(0.03) | **5.31E-04** | 3 | 169480204 | A | region is 2193 bp downstream of TERC (-) |
| rs12696304 | 0.14(0.05) | **3.63E-03** | 0.09(0.05) | 8.60E-02 | 0.12(0.04) | **1.01E-03** | 3 | 169481271 | C | region is 1126 bp downstream of TERC (-) |
| rs2068178 | 0.12(0.08) | 1.29E-01 | 0.24(0.08) | **2.13E-03** | 0.18(0.06) | **1.15E-03** | 3 | 169485639 | A | contained within ACTRT3 (-) |
| rs9822885 | 0.15(0.05) | **1.35E-03** | 0.09(0.05) | 7.96E-02 | 0.12(0.03) | **4.12E-04** | 3 | 169486144 | A | intron of ACTRT3 (-) |
| rs3821383 | 0.15(0.05) | **1.39E-03** | 0.08(0.05) | 8.84E-02 | 0.12(0.03) | **4.82E-04** | 3 | 169489946 | A | region is 906 bp upstream of MYNN (+) |
| rs10936599 | -0.19(0.05) | **3.93E-05** | -0.16(0.05) | **9.15E-04** | -0.18(0.03) | **1.32E-07** | 3 | 169492101 | A | contained within MYNN (+) |
| rs1317082 | -0.2(0.05) | **1.76E-05** | -0.16(0.05) | **9.69E-04** | -0.18(0.03) | **6.79E-08** | 3 | 169497585 | G | intron of MYNN (+) |
| rs3772188 | 0.1(0.09) | 2.63E-01 | 0.21(0.09) | **1.49E-02** | 0.16(0.06) | **1.16E-02** | 3 | 169504947 | A | contained within MYNN (+) |
| rs13069553 | -0.2(0.05) | **1.57E-05** | -0.16(0.05) | **9.54E-04** | -0.18(0.03) | **6.00E-08** | 3 | 169508272 | G | region is 767 bp downstream of MYNN (+) |
| rs7633750 | 0.17(0.05) | **6.24E-04** | 0.09(0.05) | 7.16E-02 | 0.13(0.03) | **1.99E-04** | 3 | 169509244 | G | region is 1739 bp downstream of MYNN (+) |
| rs7621631 | -0.2(0.05) | **2.00E-05** | -0.16(0.05) | **8.30E-04** | -0.18(0.03) | **6.35E-08** | 3 | 169512145 | A | intron of LRRC34 (-) |
| rs10936600 | -0.2(0.05) | **2.35E-05** | -0.16(0.05) | **6.79E-04** | -0.18(0.03) | **5.95E-08** | 3 | 169514585 | T | contained within LRRC34 (-) |
| rs6793295 | 0.16(0.05) | **9.40E-04** | 0.09(0.05) | 6.95E-02 | 0.13(0.03) | **2.62E-04** | 3 | 169518455 | A | contained within LRRC34 (-) , intron of LRRC34 (-) |
| rs10936601 | 0.16(0.05) | **1.13E-03** | 0.09(0.05) | 6.00E-02 | 0.13(0.03) | **2.56E-04** | 3 | 169528449 | G | intron of LRRC34 (-) |
| rs9878797 | 0.12(0.05) | **2.28E-02** | 0.07(0.05) | 1.66E-01 | 0.09(0.04) | **9.20E-03** | 3 | 169529958 | A | intron of LRRC34 (-) |
| rs10936602 | 0.18(0.05) | **8.86E-05** | 0.09(0.05) | 6.78E-02 | 0.14(0.03) | **3.90E-05** | 3 | 169536637 | A | region is 3072 bp upstream of LRRIQ4 (+) |
| rs16854409 | 0.16(0.12) | 2.02E-01 | -0.23(0.14) | 1.08E-01 | -0.01(0.09) | 9.17E-01 | 3 | 169538575 | A | region is 1134 bp upstream of LRRIQ4 (+) |
| rs16854411 | 0.16(0.12) | 2.02E-01 | -0.23(0.14) | 9.36E-02 | -0.01(0.09) | 8.73E-01 | 3 | 169540184 | G | contained within LRRIQ4 (+) |
| rs1920119 | 0.12(0.05) | **2.05E-02** | 0.09(0.05) | 8.76E-02 | 0.1(0.04) | **4.23E-03** | 3 | 169540397 | A | contained within LRRIQ4 (+) |
| rs10936603 | 0.17(0.05) | **2.19E-04** | 0.09(0.05) | 5.84E-02 | 0.14(0.03) | **6.25E-05** | 3 | 169545652 | C | intron of LRRIQ4 (+) |
| rs12486767 | 0.09(0.05) | 7.15E-02 | 0.09(0.05) | 8.65E-02 | 0.09(0.04) | **1.28E-02** | 3 | 169550759 | G | intron of LRRIQ4 (+) |
| rs9833035 | 0.12(0.04) | **7.71E-03** | 0.08(0.05) | 9.58E-02 | 0.1(0.03) | **2.01E-03** | 3 | 169553498 | C | intron of LRRIQ4 (+) |
| rs3732451 | 0.09(0.05) | 6.82E-02 | 0.1(0.05) | 5.97E-02 | 0.09(0.04) | **8.75E-03** | 3 | 169558025 | G | contained within LRRC31 (-) |
| rs12489230 | 0.15(0.04) | **1.23E-03** | 0.06(0.05) | 2.13E-01 | 0.1(0.03) | **1.30E-03** | 3 | 169558821 | G | intron of LRRC31 (-) |
| rs12696310 | 0.19(0.13) | 1.44E-01 | -0.23(0.15) | 1.21E-01 | 0.01(0.1) | 9.49E-01 | 3 | 169563001 | A | intron of LRRC31 (-) |
| rs13074500 | 0.15(0.04) | **1.12E-03** | 0.06(0.05) | 1.93E-01 | 0.11(0.03) | **1.07E-03** | 3 | 169565571 | A | intron of LRRC31 (-) |
| rs35923425 | 0.23(0.09) | **1.64E-02** | -0.08(0.09) | 3.53E-01 | 0.06(0.07) | 3.31E-01 | 3 | 169569432 | C | contained within LRRC31 (-) |
| rs11709840 | 0.13(0.05) | **4.64E-03** | 0.06(0.05) | 2.02E-01 | 0.1(0.03) | **3.31E-03** | 3 | 169570241 | A | intron of LRRC31 (-) |
| rs16854469 | 0.23(0.09) | **1.64E-02** | -0.07(0.09) | 4.20E-01 | 0.07(0.06) | 2.92E-01 | 3 | 169571145 | C | intron of LRRC31 (-) |
| rs3732452 | 0.04(0.1) | 6.58E-01 | 0.1(0.1) | 2.89E-01 | 0.07(0.07) | 2.86E-01 | 3 | 169572621 | A | contained within LRRC31 (-) |
| rs11919269 | 0.13(0.05) | **3.64E-03** | 0.07(0.05) | 1.68E-01 | 0.1(0.03) | **2.20E-03** | 3 | 169574465 | A | intron of LRRC31 (-) |
| rs7647589 | 0.08(0.05) | 1.17E-01 | 0.1(0.05) | 6.46E-02 | 0.09(0.04) | **1.60E-02** | 3 | 169582223 | A | intron of LRRC31 (-) |
| rs9841073 | -0.04(0.06) | 5.54E-01 | -0.08(0.06) | 1.76E-01 | -0.06(0.04) | 1.69E-01 | 3 | 169589918 | A | region is 2194 bp upstream of LRRC31 (-) |

β (SE): effect size (standard error); EA: effect allele; ^#^: hg19 genome build

**Table S5.** Pairwise Linkage Disequilibrium measures (R^2^) between the top six genetic variants in three ethnic groups. (A) Newborn genotype. (B) Maternal genotype. Genetic variants are ordered by their genomic positions.

| (A) Newborn Genotype | | | | | | | | | | | | |  |
| --- | --- | --- | --- | --- | --- | --- | --- | --- | --- | --- | --- | --- | --- |
| Chinese |  | | | | | | | | | | | |  |
| Marker | rs12638862 | | rs10936599 | | rs1317082 | | rs13069553 | | rs7621631 | | rs10936600 | |  |
| rs12638862 | 1.00 | |  | |  | |  | |  | |  | |  |
| rs10936599 | 0.93 | | 1.00 | |  | |  | |  | |  | |  |
| rs1317082 | 0.93 | | 1.00 | | 1.00 | |  | |  | |  | |  |
| rs13069553 | 0.92 | | 0.99 | | 0.99 | | 1.00 | |  | |  | |  |
| rs7621631 | 0.92 | | 0.99 | | 0.99 | | 0.99 | | 1.00 | |  | |  |
| rs10936600 | 0.92 | | 0.99 | | 0.99 | | 0.99 | | 1.00 | | 1.00 | |  |
| Malay |  | | | | | | | | | | | |  |
| Marker | rs12638862 | | rs10936599 | | rs1317082 | | rs13069553 | | rs7621631 | | rs10936600 | |  |
| rs12638862 | 1.00 | |  | |  | |  | |  | |  | |  |
| rs10936599 | 0.84 | | 1.00 | |  | |  | |  | |  | |  |
| rs1317082 | 0.84 | | 1.00 | | 1.00 | |  | |  | |  | |  |
| rs13069553 | 0.83 | | 0.99 | | 0.99 | | 1.00 | |  | |  | |  |
| rs7621631 | 0.83 | | 0.99 | | 0.99 | | 1.00 | | 1.00 | |  | |  |
| rs10936600 | 0.82 | | 0.98 | | 0.98 | | 0.99 | | 0.99 | | 1.00 | |  |
| Indian |  | | | | | | | | | | | |  |
| Marker | rs12638862 | | rs10936599 | | rs1317082 | | rs13069553 | | rs7621631 | | rs10936600 | |  |
| rs12638862 | 1.00 | |  | |  | |  | |  | |  | |  |
| rs10936599 | 0.89 | | 1.00 | |  | |  | |  | |  | |  |
| rs1317082 | 0.84 | | 0.95 | | 1.00 | |  | |  | |  | |  |
| rs13069553 | 0.84 | | 0.95 | | 1.00 | | 1.00 | |  | |  | |  |
| rs7621631 | 0.84 | | 0.95 | | 1.00 | | 1.00 | | 1.00 | |  | |  |
| rs10936600 | 0.84 | | 0.95 | | 1.00 | | 1.00 | | 1.00 | | 1.00 | |  |
| (B) Maternal Genotype | | | | | | | | | | | |  | |
| Chinese | |  | | | | | | | | | | | |
| Marker | | rs12638862 | | rs10936599 | | rs1317082 | | rs13069553 | | rs7621631 | | rs10936600 | |
| rs12638862 | | 1.00 | |  | |  | |  | |  | |  | |
| rs10936599 | | 0.90 | | 1.00 | |  | |  | |  | |  | |
| rs1317082 | | 0.90 | | 1.00 | | 1.00 | |  | |  | |  | |
| rs13069553 | | 0.90 | | 1.00 | | 1.00 | | 1.00 | |  | |  | |
| rs7621631 | | 0.90 | | 0.99 | | 0.99 | | 0.99 | | 1.00 | |  | |
| rs10936600 | | 0.90 | | 0.99 | | 0.99 | | 0.99 | | 1.00 | | 1.00 | |
| Malay | |  | | | | | | | | | | | |
| Marker | | rs12638862 | | rs10936599 | | rs1317082 | | rs13069553 | | rs7621631 | | rs10936600 | |
| rs12638862 | | 1.00 | |  | |  | |  | |  | |  | |
| rs10936599 | | 0.88 | | 1.00 | |  | |  | |  | |  | |
| rs1317082 | | 0.88 | | 1.00 | | 1.00 | |  | |  | |  | |
| rs13069553 | | 0.86 | | 0.98 | | 0.98 | | 1.00 | |  | |  | |
| rs7621631 | | 0.86 | | 0.98 | | 0.98 | | 1.00 | | 1.00 | |  | |
| rs10936600 | | 0.86 | | 0.98 | | 0.98 | | 1.00 | | 1.00 | | 1.00 | |
| Indian | |  | | | | | | | | | | | |
| Marker | | rs12638862 | | rs10936599 | | rs1317082 | | rs13069553 | | rs7621631 | | rs10936600 | |
| rs12638862 | | 1.00 | |  | |  | |  | |  | |  | |
| rs10936599 | | 0.90 | | 1.00 | |  | |  | |  | |  | |
| rs1317082 | | 0.84 | | 0.93 | | 1.00 | |  | |  | |  | |
| rs13069553 | | 0.83 | | 0.92 | | 0.98 | | 1.00 | |  | |  | |
| rs7621631 | | 0.83 | | 0.92 | | 0.98 | | 1.00 | | 1.00 | |  | |
| rs10936600 | | 0.82 | | 0.92 | | 0.98 | | 1.00 | | 1.00 | | 1.00 | |

**Table S6.** Linear regression results between maternal telomere length and antenatal maternal factors

| Category | Variables | N | Main Model  (Adjusted for age, ethnicity and DNAExtn) | | Additional Model  (Adjusted for age, ethnicity, DNAExtn and GDM status) | |
| --- | --- | --- | --- | --- | --- | --- |
|  |  |  | β (95%CI) | P Value | β (95%CI) | P Value |
| Maternal Mental Health^a^ | EPDS Score | 870 | 0.05(-0.02,0.11) | 1.35E-01 | 0.06(-0.01,0.12) | 7.20E-02 |
|  | STAI State Score | 849 | -0.01(-0.08,0.05) | 7.36E-01 | -0.01(-0.07,0.06) | 8.11E-01 |
|  | STAI Trait Score | 846 | 0.00(-0.07,0.06) | 9.07E-01 | 0.00(-0.06,0.06) | 9.98E-01 |
| Maternal Adiposity | Pre-pregnancy BMI (kg/m^2^) | 824 | -0.02(-0.09,0.05) | 5.87E-01 | 0.01(-0.06,0.08) | 8.10E-01 |
|  | Gestation Weight Gain^a^ (kg) | 822 | 0.05(-0.02,0.11) | 1.77E-01 | 0.04(-0.02,0.11) | 2.20E-01 |
|  | Height (cm) | 882 | 0.02(-0.04,0.09) | 4.76E-01 | 0.02(-0.04,0.09) | 5.04E-01 |
| Maternal Blood Pressure^a^ | Systolic BP (mmHg) | 751 | -0.01(-0.08,0.06) | 7.58E-01 | -0.01(-0.08,0.06) | 7.65E-01 |
|  | Diastolic BP (mmHg) | 751 | -0.02(-0.09,0.04) | 5.05E-01 | -0.02(-0.09,0.05) | 5.59E-01 |
| Maternal Plasma Glucose Concentration^a^ | Fasting Glucose (mmol/L) | 854 | 0.02(-0.04,0.08) | 5.04E-01 | 0.05(-0.02,0.11) | 1.35E-01 |
|  | 2-h Post-load Glucose (mmol/L) | 854 | -0.09(-0.15,-0.02) | **6.62E-03*** | --- | --- |
|  | Gestational Diabetes Mellitus  0: Non-GDM  1: GDM | 698  156 | -0.27(-0.43,-0.10) | **1.64E-03*** | --- | **---** |
| Maternal Plasma Fatty Acid^a^ | Total SFA% | 787 | -0.01(-0.08,0.06) | 7.54E-01 | -0.01(-0.08,0.06) | 8.09E-01 |
|  | Total MUFA% | 787 | 0.00(-0.07,0.08) | 9.03E-01 | -0.01(-0.08,0.06) | 7.87E-01 |
|  | Total n-6 PUFA% | 787 | -0.03(-0.10,0.04) | 3.56E-01 | -0.03(-0.10,0.05) | 4.78E-01 |
|  | LA% | 787 | -0.06(-0.13,0.01) | 7.31E-02 | -0.05(-0.12,0.02) | 1.50E-01 |
|  | DGLA% | 786 | 0.02(-0.05,0.09) | 5.08E-01 | 0.01(-0.06,0.09) | 6.80E-01 |
|  | AA% | 787 | 0.04(-0.03,0.11) | 2.94E-01 | 0.04(-0.04,0.11) | 3.23E-01 |
|  | Total n-3 PUFA% | 787 | 0.08(0.01,0.15) | **3.23E-02*** | 0.08(0.00,0.15) | **3.85E-02*** |
|  | DHA% | 787 | 0.06(-0.01,0.14) | 7.13E-02 | 0.06(-0.01,0.13) | 8.31E-02 |
|  | DPA% | 787 | 0.04(-0.03,0.11) | 3.16E-01 | 0.03(-0.04,0.10) | 3.70E-01 |
|  | EPA% | 787 | 0.05(-0.02,0.12) | 1.74E-01 | 0.05(-0.02,0.12) | 1.48E-01 |
|  | n-6:n-3 PUFA Ratio | 787 | -0.08(-0.15,-0.01) | **2.19E-02*** | -0.08(-0.15,-0.01) | **2.67E-02*** |
| Maternal Plasma Vitamins and Metabolites^a^ | Vitamin B6 (nmol/L), log_10_ | 780 | 0.02(-0.05,0.09) | 5.84E-01 | 0.03(-0.04,0.1) | 4.65E-01 |
|  | Vitamin B12 (pg/ml), log_10_ | 789 | 0.05(-0.03,0.12) | 2.02E-01 | 0.04(-0.03,0.12) | 2.27E-01 |
|  | Folate (ng/ml), log_10_ | 789 | -0.08(-0.15,-0.01) | **2.27E-02*** | -0.07(-0.14,0.01) | 8.11E-02 |
|  | Vitamin D_3_ (nmol/L) | 751 | 0.00(-0.07,0.08) | 9.37E-01 | -0.01(-0.08,0.07) | 8.82E-01 |
|  | Betaine (µmol/L), log_10_ | 780 | 0.00(-0.07,0.07) | 9.99E-01 | 0.02(-0.05,0.09) | 6.23E-01 |
|  | Choline (µmol/L), log_10_ | 780 | 0.00(-0.07,0.07) | 9.43E-01 | 0.01(-0.06,0.08) | 8.08E-01 |
|  | Homocysteine (µmol/L), log_10_ | 780 | -0.04(-0.11,0.03) | 2.57E-01 | -0.04(-0.11,0.03) | 2.20E-01 |
| Maternal Plasma Protein Biomarkers^a^ | Adiponectin (pg/ml), log_10_ | 892 | -0.01(-0.07,0.06) | 7.95E-01 | -0.04(-0.11,0.02) | 2.14E-01 |
|  | Leptin (pg/ml), log_10_ | 876 | 0.02(-0.05,0.08) | 6.24E-01 | 0.01(-0.06,0.08) | 7.59E-01 |
|  | C-Reactive Protein (ng/ml), log_10_ | 892 | -0.01(-0.07,0.06) | 7.86E-01 | -0.01(-0.07,0.06) | 8.67E-01 |
|  | PAI-1 (pg/ml), log_10_ | 885 | -0.07(-0.13,-0.01) | **2.40E-02*** | -0.08(-0.14,-0.02) | **1.46E-02*** |
|  | IGF1 (pg/ml), log_10_ | 892 | 0.02(-0.04,0.08) | 4.90E-01 | 0.02(-0.05,0.08) | 5.73E-01 |
|  | IGF2 (pg/ml), log_10_ | 811 | 0.07(0.01,0.14) | **3.25E-02*** | 0.07(0.01,0.14) | **3.28E-02*** |
|  | IGFBP1 (ng/ml), log_10_ | 892 | 0.01(-0.05,0.07) | 8.05E-01 | -0.02(-0.08,0.05) | 6.21E-01 |
|  | IGFBP3 (ng/ml), log_10_ | 891 | -0.02(-0.09,0.04) | 4.57E-01 | -0.04(-0.1,0.02) | 2.00E-01 |
|  | IGFBP4 (ng/ml), log_10_ | 871 | -0.03(-0.09,0.04) | 3.91E-01 | -0.04(-0.11,0.02) | 1.83E-01 |
|  | IGFBP7 (ng/ml), log_10_ | 892 | 0.02(-0.04,0.08) | 5.78E-01 | 0.01(-0.05,0.08) | 6.53E-01 |
| Socio-economic  Status  (SES) | Maternal Education  1: Secondary and below  2: Post-secondary  3: University | 262  309  309 | 0.09(0.01,0.17) | **2.73E-02*** | 0.11(0.03,0.19) | **9.76E-03*** |
|  | Household Income  1: ≤S$1999  2: S$2000-5999  3:≥S$6000 | 124  457  250 | 0.02(-0.09,0.12) | 7.22E-01 | 0.02(-0.08,0.13) | 6.48E-01 |
| Maternal Smoking Status | Before pregnancy  0: No  1: Yes | 773  114 | -0.06(-0.26,0.14) | 5.51E-01 | -0.09(-0.28,0.11) | 3.94E-01 |
|  | During pregnancy  0: No  1: Yes | 746  35 | -0.38(-0.71,-0.05) | **2.42E-02*** | -0.4(-0.72,-0.07) | **1.71E-02*** |
| Maternal Alcohol Consumption | Before pregnancy  0: No  1: Yes | 574  315 | 0.00(-0.14,0.14) | 9.94E-01 | -0.01(-0.15,0.13) | 8.82E-01 |
|  | During pregnancy  0: No  1: Yes | 850  20 | 0.21(-0.21,0.63) | 3.34E-01 | 0.19(-0.22,0.61) | 3.61E-01 |
| Parity | 0: Primiparous  1: Multiparous | 406  486 | -0.05(-0.18,0.08) | 4.79E-01 | -0.05(-0.19,0.08) | 4.46E-01 |

P<0.05 *; P<0.001**;  ^a^: measured at 26-28 weeks’ pregnancy; DNAExtn: DNA extraction method; β: effect size; CI: confidence interval; log_10_: those variables with skewed distribution are log10-transformed for linear regression analysis; EPDS: Edinburgh Postnatal Depression Scale; STAI: The State-Trait Anxiety Inventory; SFA: Saturated Fatty Acid; MUFA: Mono-Unsaturated Fatty Acid; PUFA: Poly-Unsaturated Fatty Acid; LA: Linoleic Acid; DGLA: Dihomo-Gamma-Linolenic Acid; AA: Arachidonic Acid; DHA: Docosahexaenoic Acid; DPA: Docosapentaenoic Acid; EPA: Eicosapentaenoic Acid; IGF: Insulin-like Growth Factor; IGFBP: Insulin-like Growth Factor Binding Protein; PAI-1: Plasminogen Activator Inhibitor-1

**Table S7.** The results of sensitivity analysis after adding DNA storage time and sample collection month in the best multivariate models of newborn telomere length using all, male only and female only subjects. Related to Table 3.

| Variable | All (N=721) | | | Male (N=378) | | Female (N=405) | |
| --- | --- | --- | --- | --- | --- | --- | --- |
|  | β (95%CI) | | P Value | β (95%CI) | P Value | β (95%CI) | P Value |
| Sex  Male  Female | | Ref  0.44(0.30,0.57) | Ref  **9.14E-10***** | --- | --- | --- | --- |
|  | |  |  |  |  |  |  |
| Ethnicity  Chinese  Malay  Indian | | Ref  -0.17(-0.34,0.00)  -0.22(-0.43,-0.01) | Ref  **4.92E-02***  **3.87E-02*** | Ref  -0.10(-0.32,0.13)  -0.22(-0.52,0.08) | Ref  4.02E-01  1.43E-01 | Ref  -0.34(-0.56,-0.11)  -0.19(-0.46,0.08) | Ref  **3.86E-03***  1.71E-01 |
| Newborn *LRRC34*- rs10936600  (0-AA 1-AT 2-TT) | | -0.19(-0.29,-0.09) | **3.29E-04**** | -0.23(-0.38,-0.09) | **1.30E-03*** | -0.13(-0.27,0.00) | 5.01E-02 |
| Maternal Telomere Length (T/S) | | 0.12(0.05,0.19) | **1.18E-03**** | 0.10(0.00,0.20) | **4.77E-02*** | 0.16(0.07,0.25) | **8.68E-04**** |
| Paternal Age (years) | | 0.14(0.07,0.21) | **1.32E-04**** | 0.19(0.10,0.29) | **5.94E-05**** | --- | **---** |
| Plasma Fasting Glucose (mmol/L) | | -0.08(-0.15,-0.02) | **1.60E-02*** | -0.16(-0.26,-0.06) | **2.40E-03*** | --- | --- |
| Plasma IGFBP3 (ng/ml), log_10_ | | -0.08(-0.15,-0.01) | **3.35E-02*** | -0.18(-0.28,-0.08) | **3.59E-04**** | --- | **---** |
| STAI Trait Score | | --- | --- | --- | **---** | -0.14(-0.23,-0.05) | **2.74E-03*** |
| DNA Storage Time | | -0.15(-0.33,0.03) | 1.12E-01 | -0.14(-0.39,0.12) | 2.87E-01 | -0.19(-0.43,0.05) | 1.30E-01 |
| Sample Collection Month  Jan-Mar  Apr-Jun  Jul-Sep  Oct-Dec | | Ref  -0.14(-0.35,0.08)  0.06(-0.15,0.26)  0.10(-0.08,0.28) | 2.12E-01  5.89E-01  2.87E-01 | Ref  0.00(-0.30,0.29)  0.03(-0.23,0.30)  0.06(-0.19,0.32) | Ref  9.96E-01  8.04E-01  6.21E-01 | Ref  -0.04(-0.31,0.23)  0.06(-0.21,0.33)  0.17(-0.06,0.41) | Ref  7.60E-01  6.81E-01  1.53E-01 |

P<0.05*; P<0.001**; P<1.00E-06***; Underscore: borderline P value

**Table S8.** The results of sensitivity analysis after further adjustment for DNA storage time and sample collection month in the association studies between maternal telomere length and antenatal maternal factors (supplementary model)

| Category | Variables | N | Main Model (Table S6)  (Adjusted for age, ethnicity and DNAExtn) | | Supplementary Model  (Adjusted for age, ethnicity, DNAExtn, DNA storage time and sample collection month) | |
| --- | --- | --- | --- | --- | --- | --- |
|  |  |  | β (95%CI) | P Value | β (95%CI) | P Value |
| Maternal Plasma Glucose Concentration | 2-h Post-load Glucose (mmol/L) | 854 | -0.09(-0.15,-0.02) | **6.62E-03*** | -0.07(-0.13,0) | **4.29E-02*** |
|  | Gestational Diabetes Mellitus  0: Non-GDM  1: GDM | 698  156 | -0.27(-0.43,-0.10) | **1.64E-03*** | -0.23(-0.39,-0.07) | **5.16E-03*** |
| Maternal Plasma Fatty Acid | Total n-3 PUFA% | 787 | 0.08(0.01,0.15) | **3.23E-02*** | 0.07(0.00,0.14) | 5.56E-02 |
|  | n-6:n-3 PUFA Ratio | 787 | -0.08(-0.15,-0.01) | **2.19E-02*** | -0.07(-0.14,0.00) | **4.22E-02*** |
| Maternal Plasma Vitamins and Metabolites | Folate (ng/ml), log_10_ | 789 | -0.08(-0.15,-0.01) | **2.27E-02*** | -0.07(-0.14,0.00) | 5.77E-02 |
| Maternal Plasma Protein Biomarkers | PAI-1 (pg/ml), log_10_ | 885 | -0.07(-0.13,-0.01) | **2.40E-02*** | -0.05(-0.11,0.02) | 1.42E-01 |
|  | IGF2 (pg/ml), log_10_ | 811 | 0.07(0.01,0.14) | **3.25E-02*** | 0.05(-0.01,0.12) | 9.49E-02 |
| Socio-economic  Status  (SES) | Maternal Education  1: Secondary and below  2: Post-secondary  3: University | 262  309  309 | 0.09(0.01,0.17) | **2.73E-02*** | 0.12(0.04,0.20) | **3.05E-03*** |
| Maternal Smoking Status | During pregnancy  0: No  1: Yes | 746  35 | -0.38(-0.71,-0.05) | **2.42E-02*** | -0.39(-0.71,-0.07) | **1.68E-02*** |

P<0.05*; Underscore: borderline P value.

**Table S9.** The genetic variants in a strong Linkage Disequilibrium (R^2^>0.85) with rs10936600 (query SNP) using LDproxy and CHB population (https://analysistools.nci.nih.gov/LDlink). Top six genetic variants in the meta-analysis results are highlighted as grey. The variant rs2293607 (*TERC*:500B downstream variant) has been reported that the T allele (correlated to A allele in rs10936600) is associated with an increase in *TERC* expression and telomere length [56].

| rsID | Chr:Position | Alleles | MAF | Distance | D’ | R^2^ | Correlated Alleles |
| --- | --- | --- | --- | --- | --- | --- | --- |
| rs10936600 | chr3:169514585 | (A/T) | 0.4078 | 0 | 1 | 1 | A=A,T=T |
| rs139385377 | chr3:169513706 | (AA/-) | 0.4078 | -879 | 1 | 1 | A=AA,T=- |
| rs7643115 | chr3:169512241 | (G/A) | 0.4078 | -2344 | 1 | 1 | A=G,T=A |
| rs7621631 | chr3:169512145 | (C/A) | 0.4078 | -2440 | 1 | 1 | A=C,T=A |
| rs13069553 | chr3:169508272 | (A/G) | 0.4078 | -6313 | 1 | 1 | A=A,T=G |
| rs71882452 | chr3:169522390 | (AT/-) | 0.4078 | 7805 | 1 | 1 | A=AT,T=- |
| rs11711621 | chr3:169524016 | (C/T) | 0.4078 | 9431 | 1 | 1 | A=C,T=T |
| rs3796145 | chr3:169524862 | (A/C) | 0.4078 | 10277 | 1 | 1 | A=A,T=C |
| rs28626343 | chr3:169526272 | (T/C) | 0.4078 | 11687 | 1 | 1 | A=T,T=C |
| rs3772190 | chr3:169500487 | (G/A) | 0.4078 | -14098 | 1 | 1 | A=G,T=A |
| rs35379604 | chr3:169495947 | (ATC/-) | 0.4078 | -18638 | 1 | 1 | A=ATC,T=- |
| rs10936599 | chr3:169492101 | (C/T) | 0.4078 | -22484 | 1 | 1 | A=C,T=T |
| rs34847803 | chr3:169492016 | (-/A) | 0.4078 | -22569 | 1 | 1 | A=-,T=A |
| rs12637184 | chr3:169487437 | (G/A) | 0.4078 | -27148 | 1 | 1 | A=G,T=A |
| rs35446936 | chr3:169486508 | (G/A) | 0.4078 | -28077 | 1 | 1 | A=G,T=A |
| rs1317082 | chr3:169497585 | (A/G) | 0.4029 | -17000 | 1 | 0.9801 | A=A,T=G |
| rs3950296 | chr3:169493283 | (C/G) | 0.4029 | -21302 | 1 | 0.9801 | A=C,T=G |
| rs2251795 | chr3:169491729 | (T/A) | 0.4029 | -22856 | 1 | 0.9801 | A=T,T=A |
| **rs2293607** | chr3:169482335 | (T/C) | 0.4029 | -32250 | 1 | 0.9801 | A=T,T=C |
| rs12638862 | chr3:169477506 | (A/G) | 0.3883 | -37079 | 1 | 0.9221 | A=A,T=G |
| rs11344572 | chr3:169527746 | (T/-) | 0.3835 | 13161 | 1 | 0.9034 | A=T,T=- |

MAF: minor allele frequency; D’: an indicator of allelic segregation for two genetic variants

R^2^: a measure of correlation of alleles for two genetic variants.

**Table S10.** The association of candidate genes (*TERT, RTEL1, OBFC1, NAF1, ZNF208* and *ACYP2*) in the GWAS results of newborn and maternal telomere lengths and the meta-analysis results. Significant P values (P<0.05) are highlighted as bold.

| **rsID** | **Newborn TL** | | **Maternal TL** | | **Meta-analysis** | | **Annotation** | | | |
| --- | --- | --- | --- | --- | --- | --- | --- | --- | --- | --- |
|  | **β(SE)** | **P Value** | **β(SE)** | **P Value** | **β(SE)** | **P Value** | **Chr** | **Position^#^** | **EA** | **Features** |
| rs2853690 | 0.27(0.17) | 1.26E-01 | 0.33(0.19) | 8.93E-02 | 0.3(0.13) | **2.29E-02** | 5 | 1253744 | A | contained within TERT (-) |
| rs2736122 | 0.21(0.08) | **6.12E-03** | 0.02(0.08) | 8.15E-01 | 0.12(0.06) | **2.83E-02** | 5 | 1257621 | A | intron of TERT (-) |
| rs2075786 | 0.1(0.05) | 5.96E-02 | -0.01(0.06) | 8.17E-01 | 0.05(0.04) | 2.19E-01 | 5 | 1266310 | G | intron of TERT (-) |
| rs10069690 | 0.02(0.05) | 6.66E-01 | 0.09(0.06) | 1.08E-01 | 0.06(0.04) | 1.57E-01 | 5 | 1279790 | A | intron of TERT (-) |
| rs2736100 | 0.07(0.04) | 1.13E-01 | 0.1(0.05) | **2.87E-02** | 0.09(0.03) | **7.88E-03** | 5 | 1286516 | C | intron of TERT (-) |
| rs2853677 | 0.05(0.04) | 2.51E-01 | 0.18(0.05) | **1.85E-04** | 0.11(0.03) | **6.39E-04** | 5 | 1287194 | G | intron of TERT (-) |
| rs2853676 | 0.02(0.06) | 7.37E-01 | 0.1(0.06) | 6.69E-02 | 0.06(0.04) | 1.27E-01 | 5 | 1288547 | A | intron of TERT (-) |
| rs2853672 | -0.05(0.04) | 2.28E-01 | -0.1(0.05) | **2.80E-02** | -0.08(0.03) | **1.75E-02** | 5 | 1292983 | A | intron of TERT (-) |
| rs3761124 | 0.01(0.05) | 7.88E-01 | -0.04(0.05) | 4.66E-01 | -0.01(0.03) | 7.52E-01 | 20 | 62288752 | G | region is 410 bp upstream of RTEL1 (+) |
| rs6122022 | 0.01(0.07) | 8.90E-01 | 0.1(0.08) | 1.97E-01 | 0.05(0.05) | 3.28E-01 | 20 | 62289595 | A | contained within RTEL1 (+) , contained within RTEL1-TNFRSF6B (+) |
| rs3848668 | 0.02(0.19) | 8.99E-01 | 0.39(0.21) | 6.37E-02 | 0.19(0.14) | 1.79E-01 | 20 | 62293272 | G | contained within RTEL1 (+) , contained within RTEL1-TNFRSF6B (+) |
| rs2297434 | 0(0.06) | 9.72E-01 | -0.01(0.06) | 9.08E-01 | 0(0.04) | 9.55E-01 | 20 | 62294015 | A | intron of RTEL1 (+), intron of RTEL1-TNFRSF6B (+) |
| rs6011002 | 0.02(0.19) | 8.99E-01 | 0.39(0.21) | 6.29E-02 | 0.19(0.14) | 1.78E-01 | 20 | 62297802 | G | intron of RTEL1 (+), intron of RTEL1-TNFRSF6B (+) |
| rs6011011 | 0.02(0.19) | 8.99E-01 | 0.39(0.21) | 6.37E-02 | 0.19(0.14) | 1.79E-01 | 20 | 62299578 | A | intron of RTEL1 (+), intron of RTEL1-TNFRSF6B (+) |
| rs2297437 | 0(0.06) | 9.65E-01 | -0.07(0.06) | 2.23E-01 | -0.04(0.04) | 3.73E-01 | 20 | 62305274 | A | intron of RTEL1 (+), intron of RTEL1-TNFRSF6B (+) |
| rs6089956 | 0.06(0.05) | 2.31E-01 | 0.03(0.05) | 5.11E-01 | 0.05(0.04) | 1.89E-01 | 20 | 62308364 | A | intron of RTEL1 (+), intron of RTEL1-TNFRSF6B (+) |
| rs6010620 | -0.02(0.05) | 7.12E-01 | -0.07(0.05) | 1.36E-01 | -0.05(0.03) | 1.89E-01 | 20 | 62309839 | G | intron of RTEL1 (+), intron of RTEL1-TNFRSF6B (+) |
| rs4809324 | -0.07(0.07) | 2.86E-01 | -0.02(0.07) | 7.63E-01 | -0.05(0.05) | 3.25E-01 | 20 | 62318220 | G | intron of RTEL1 (+), intron of RTEL1-TNFRSF6B (+) |
| rs6062302 | -0.03(0.05) | 5.78E-01 | -0.08(0.05) | 9.78E-02 | -0.05(0.03) | 1.17E-01 | 20 | 62320968 | G | contained within RTEL1 (+) , contained within RTEL1-TNFRSF6B (+) |
| rs3208008 | -0.03(0.05) | 5.43E-01 | -0.06(0.05) | 1.95E-01 | -0.05(0.03) | 1.78E-01 | 20 | 62326110 | C | contained within RTEL1 (+) , contained within RTEL1-TNFRSF6B (+) |
| rs11191839 | 0.01(0.05) | 7.79E-01 | -0.01(0.05) | 8.16E-01 | 0(0.03) | 9.71E-01 | 10 | 105634895 | G | region is 2422 bp downstream of OBFC1 (-) |
| rs2902638 | 0.05(0.05) | 3.85E-01 | -0.02(0.06) | 7.30E-01 | 0.02(0.04) | 6.90E-01 | 10 | 105636989 | G | region is 328 bp downstream of OBFC1 (-) |
| rs4917405 | -0.01(0.13) | 9.50E-01 | -0.1(0.12) | 4.01E-01 | -0.06(0.09) | 5.15E-01 | 10 | 105638232 | A | contained within OBFC1 (-) |
| rs911547 | -0.08(0.11) | 4.75E-01 | -0.09(0.11) | 4.23E-01 | -0.08(0.08) | 2.84E-01 | 10 | 105639421 | G | contained within OBFC1 (-) |
| rs10748858 | -0.02(0.05) | 6.65E-01 | 0(0.05) | 9.50E-01 | -0.01(0.04) | 7.25E-01 | 10 | 105639514 | C | contained within OBFC1 (-) |
| rs7100920 | 0.03(0.05) | 5.19E-01 | 0.02(0.05) | 6.95E-01 | 0.02(0.03) | 4.62E-01 | 10 | 105640978 | A | contained within OBFC1 (-) |
| rs2067832 | 0.03(0.05) | 5.19E-01 | 0.02(0.05) | 6.95E-01 | 0.02(0.03) | 4.62E-01 | 10 | 105643134 | A | intron of OBFC1 (-) |
| rs3814219 | 0(0.06) | 9.93E-01 | 0.05(0.07) | 4.97E-01 | 0.02(0.05) | 6.43E-01 | 10 | 105647095 | A | intron of OBFC1 (-) |
| rs4918069 | 0.04(0.05) | 4.58E-01 | -0.01(0.06) | 8.72E-01 | 0.02(0.04) | 6.65E-01 | 10 | 105654391 | C | intron of OBFC1 (-) |
| rs2984132 | 0.03(0.05) | 5.16E-01 | 0.02(0.05) | 7.35E-01 | 0.02(0.03) | 4.83E-01 | 10 | 105657892 | A | intron of OBFC1 (-) |
| rs1265164 | -0.09(0.12) | 4.60E-01 | -0.14(0.12) | 2.42E-01 | -0.11(0.08) | 1.76E-01 | 10 | 105674854 | A | intron of OBFC1 (-) |
| rs9419958 | -0.07(0.12) | 5.58E-01 | -0.14(0.12) | 2.42E-01 | -0.1(0.08) | 2.12E-01 | 10 | 105675946 | A | intron of OBFC1 (-) |
| rs4387287 | -0.01(0.06) | 8.22E-01 | 0.02(0.06) | 6.78E-01 | 0.01(0.04) | 8.93E-01 | 10 | 105677897 | A | contained within OBFC1 (-) |
| rs1055263 | -0.16(0.06) | **5.44E-03** | -0.08(0.06) | 1.61E-01 | -0.12(0.04) | **2.85E-03** | 4 | 164045822 | A | region is 2037 bp downstream of NAF1 (-) |
| rs4691895 | -0.16(0.06) | **5.44E-03** | -0.09(0.06) | 1.39E-01 | -0.13(0.04) | **2.38E-03** | 4 | 164048199 | G | contained within NAF1 (-) |
| rs1061075 | -0.04(0.08) | 6.11E-01 | -0.03(0.08) | 7.36E-01 | -0.03(0.06) | 5.50E-01 | 4 | 164049916 | C | contained within NAF1 (-) , intron of NAF1 (-) |
| rs7693533 | -0.16(0.06) | **6.82E-03** | -0.09(0.06) | 1.44E-01 | -0.12(0.04) | **3.01E-03** | 4 | 164066710 | A | intron of NAF1 (-) |
| rs17609340 | 0.09(0.09) | 3.45E-01 | -0.07(0.1) | 5.06E-01 | 0.02(0.07) | 8.16E-01 | 4 | 164081668 | C | intron of NAF1 (-) |
| rs6536704 | -0.07(0.05) | 1.48E-01 | -0.04(0.05) | 4.29E-01 | -0.05(0.03) | 1.13E-01 | 4 | 164083616 | A | intron of NAF1 (-) |
| rs4691896 | -0.16(0.06) | **4.61E-03** | -0.1(0.06) | 9.78E-02 | -0.14(0.04) | **1.34E-03** | 4 | 164085425 | A | contained within NAF1 (-) |
| rs17609388 | 0.03(0.08) | 7.03E-01 | -0.01(0.08) | 9.46E-01 | 0.01(0.06) | 8.14E-01 | 4 | 164092042 | G | region is 3968 bp upstream of NAF1 (-) |
| rs12608935 | -0.01(0.05) | 8.07E-01 | -0.06(0.05) | 3.04E-01 | -0.03(0.04) | 3.89E-01 | 19 | 22145147 | G | region is 3749 bp downstream of ZNF208 (-) |
| rs10418985 | 0.06(0.05) | 2.23E-01 | 0.12(0.05) | **1.35E-02** | 0.09(0.03) | **9.59E-03** | 19 | 22147952 | A | region is 944 bp downstream of ZNF208 (-) |
| rs2188971 | 0.07(0.05) | 1.53E-01 | 0.11(0.05) | **2.50E-02** | 0.09(0.03) | **9.75E-03** | 19 | 22152182 | A | contained within ZNF208 (-) |
| rs10426971 | -0.04(0.05) | 3.54E-01 | -0.11(0.05) | **2.81E-02** | -0.08(0.03) | **2.73E-02** | 19 | 22158167 | A | intron of ZNF208 (-) |
| rs8103214 | 0.07(0.05) | 1.39E-01 | 0.11(0.05) | **2.31E-02** | 0.09(0.03) | **8.26E-03** | 19 | 22158992 | G | intron of ZNF208 (-) |
| rs2079063 | -0.04(0.05) | 4.92E-01 | -0.01(0.06) | 9.15E-01 | -0.02(0.04) | 5.68E-01 | 19 | 22160674 | A | intron of ZNF208 (-) |
| rs10416159 | 0.07(0.05) | 1.31E-01 | 0.12(0.05) | **1.79E-02** | 0.09(0.03) | **6.30E-03** | 19 | 22168802 | G | intron of ZNF208 (-) |
| rs1988501 | 0.07(0.05) | 1.31E-01 | 0.12(0.05) | **1.79E-02** | 0.09(0.03) | **6.30E-03** | 19 | 22175566 | A | intron of ZNF208 (-) |
| rs2359815 | 0.07(0.05) | 1.26E-01 | 0.12(0.05) | **1.70E-02** | 0.09(0.03) | **5.84E-03** | 19 | 22187760 | C | intron of ZNF208 (-) |
| rs12617094 | 0.08(0.05) | 1.54E-01 | 0.13(0.06) | **2.25E-02** | 0.1(0.04) | **8.88E-03** | 2 | 54341098 | A | region is 1311 bp upstream of ACYP2 (+) |
| rs12620359 | 0.06(0.05) | 2.60E-01 | 0.11(0.05) | **4.62E-02** | 0.08(0.04) | **2.76E-02** | 2 | 54341106 | G | region is 1303 bp upstream of ACYP2 (+) |
| rs2287641 | 0.08(0.05) | 1.57E-01 | 0.12(0.06) | **2.80E-02** | 0.1(0.04) | **1.08E-02** | 2 | 54342955 | A | intron of ACYP2 (+) |
| rs10206954 | 0.05(0.05) | 3.11E-01 | 0.1(0.05) | 7.16E-02 | 0.07(0.04) | **4.74E-02** | 2 | 54358517 | A | intron of ACYP2 (+) |
| rs10186140 | 0.05(0.05) | 3.56E-01 | 0.09(0.05) | 8.99E-02 | 0.07(0.04) | 6.54E-02 | 2 | 54362975 | G | intron of ACYP2 (+) |
| rs1421619 | 0.06(0.05) | 2.39E-01 | 0.1(0.05) | 5.73E-02 | 0.08(0.04) | **3.00E-02** | 2 | 54366449 | G | intron of ACYP2 (+) |
| rs7572559 | 0.04(0.05) | 4.56E-01 | 0.1(0.05) | 6.07E-02 | 0.07(0.04) | 6.64E-02 | 2 | 54372118 | A | intron of ACYP2 (+) |
| rs10201710 | 0.04(0.05) | 4.43E-01 | 0.09(0.05) | 9.07E-02 | 0.06(0.04) | 8.49E-02 | 2 | 54372931 | G | intron of ACYP2 (+) |
| rs13399590 | 0.05(0.05) | 3.73E-01 | 0.1(0.05) | 7.29E-02 | 0.07(0.04) | 5.98E-02 | 2 | 54382335 | G | intron of ACYP2 (+) |
| rs6545388 | -0.04(0.05) | 3.73E-01 | -0.03(0.05) | 5.40E-01 | -0.04(0.04) | 2.84E-01 | 2 | 54384927 | A | intron of ACYP2 (+) |
| rs6545389 | -0.08(0.07) | 2.89E-01 | -0.06(0.08) | 4.19E-01 | -0.07(0.05) | 1.85E-01 | 2 | 54386901 | A | intron of ACYP2 (+) |
| rs6744662 | 0.09(0.05) | 5.85E-02 | 0.09(0.05) | 8.58E-02 | 0.09(0.03) | **1.05E-02** | 2 | 54393570 | A | intron of ACYP2 (+) |
| rs1215153 | -0.02(0.05) | 7.16E-01 | -0.01(0.05) | 8.09E-01 | -0.01(0.03) | 6.68E-01 | 2 | 54402934 | G | intron of ACYP2 (+) |
| rs843677 | -0.05(0.05) | 2.64E-01 | -0.07(0.05) | 1.73E-01 | -0.06(0.03) | 8.05E-02 | 2 | 54409855 | G | intron of ACYP2 (+) |
| rs3850348 | 0.04(0.06) | 4.28E-01 | 0.04(0.06) | 4.97E-01 | 0.04(0.04) | 2.97E-01 | 2 | 54412539 | A | intron of ACYP2 (+) |
| rs13417493 | 0.09(0.05) | 7.87E-02 | 0.06(0.05) | 2.54E-01 | 0.07(0.03) | **3.98E-02** | 2 | 54420617 | A | intron of ACYP2 (+) |
| rs843671 | -0.07(0.05) | 1.46E-01 | -0.03(0.05) | 6.11E-01 | -0.05(0.03) | 1.61E-01 | 2 | 54420839 | C | intron of ACYP2 (+) |
| rs1385274 | -0.05(0.05) | 3.26E-01 | 0(0.05) | 9.41E-01 | -0.02(0.03) | 5.13E-01 | 2 | 54424537 | A | intron of ACYP2 (+) |
| rs11894205 | 0.08(0.05) | 9.41E-02 | 0.04(0.05) | 4.24E-01 | 0.06(0.03) | 7.82E-02 | 2 | 54442697 | A | intron of ACYP2 (+) |
| rs843755 | -0.05(0.05) | 3.31E-01 | 0.01(0.05) | 8.75E-01 | -0.02(0.03) | 5.52E-01 | 2 | 54448555 | C | intron of ACYP2 (+) |
| rs843653 | 0.03(0.05) | 5.56E-01 | 0.01(0.05) | 8.58E-01 | 0.02(0.03) | 5.83E-01 | 2 | 54469283 | A | intron of ACYP2 (+) |
| rs17045711 | 0.13(0.06) | **3.93E-02** | 0.02(0.07) | 7.56E-01 | 0.08(0.05) | 8.82E-02 | 2 | 54476678 | A | intron of ACYP2 (+) |
| rs843711 | 0.04(0.05) | 3.89E-01 | 0.03(0.05) | 4.67E-01 | 0.04(0.03) | 2.61E-01 | 2 | 54479117 | A | intron of ACYP2 (+) |
| rs843706 | 0.03(0.05) | 4.51E-01 | 0.04(0.05) | 4.11E-01 | 0.04(0.03) | 2.66E-01 | 2 | 54480369 | A | contained within TSPYL6 (-) , intron of ACYP2 (+) |
| rs10165485 | 0.14(0.07) | **3.17E-02** | 0.02(0.07) | 7.80E-01 | 0.08(0.05) | 7.96E-02 | 2 | 54481636 | G | contained within TSPYL6 (-) , intron of ACYP2 (+) |
| rs843704 | -0.04(0.05) | 3.73E-01 | 0.01(0.05) | 7.93E-01 | -0.02(0.03) | 6.49E-01 | 2 | 54482964 | A | contained within TSPYL6 (-) , intron of ACYP2 (+) |
| rs843688 | 0.03(0.05) | 4.91E-01 | 0.04(0.05) | 3.86E-01 | 0.04(0.03) | 2.72E-01 | 2 | 54490026 | A | intron of ACYP2 (+) |
| rs10171249 | 0.12(0.06) | 5.87E-02 | 0.1(0.07) | 1.33E-01 | 0.11(0.05) | **1.61E-02** | 2 | 54493408 | C | intron of ACYP2 (+) |
| rs12465259 | -0.02(0.05) | 7.52E-01 | -0.05(0.06) | 3.92E-01 | -0.03(0.04) | 4.10E-01 | 2 | 54498212 | G | intron of ACYP2 (+) |
| rs843743 | -0.05(0.05) | 2.74E-01 | -0.02(0.05) | 7.09E-01 | -0.04(0.03) | 3.01E-01 | 2 | 54501025 | A | intron of ACYP2 (+) |
| rs843748 | 0.12(0.05) | **2.46E-02** | 0.04(0.06) | 4.83E-01 | 0.08(0.04) | **3.31E-02** | 2 | 54502912 | A | intron of ACYP2 (+) |
| rs843713 | 0.03(0.04) | 4.53E-01 | 0.05(0.05) | 2.51E-01 | 0.04(0.03) | 1.81E-01 | 2 | 54506070 | G | intron of ACYP2 (+) |
| rs843718 | -0.06(0.05) | 2.46E-01 | -0.03(0.05) | 5.43E-01 | -0.04(0.03) | 2.08E-01 | 2 | 54508110 | G | intron of ACYP2 (+) |
| rs843719 | -0.07(0.05) | 1.69E-01 | -0.03(0.05) | 5.27E-01 | -0.05(0.03) | 1.52E-01 | 2 | 54509549 | G | intron of ACYP2 (+) |
| rs843726 | 0.12(0.09) | 1.85E-01 | -0.13(0.1) | 1.86E-01 | 0(0.07) | 9.46E-01 | 2 | 54513742 | A | intron of ACYP2 (+) |

β (SE): effect size (standard error); EA: effect allele;  ^#^: hg19 genome build
